# Mechanistic insight into the role of AUXIN RESISTANCE4 in trafficking of AUXIN1 and LIKE AUX1-2

**DOI:** 10.1093/plphys/kiad506

**Published:** 2023-09-30

**Authors:** Alison Tidy, Norliza Abu Bakar, David Carrier, Ian D Kerr, Charlie Hodgman, Malcolm J Bennett, Ranjan Swarup

**Affiliations:** Division of Plant and Crop Science, School of Biosciences, University of Nottingham, Nottingham LE12 5RD, UK; Division of Plant and Crop Science, School of Biosciences, University of Nottingham, Nottingham LE12 5RD, UK; School of Life Sciences, University of Nottingham, Queen's Medical Centre, Nottingham NG7 2UH, UK; School of Life Sciences, University of Nottingham, Queen's Medical Centre, Nottingham NG7 2UH, UK; Division of Plant and Crop Science, School of Biosciences, University of Nottingham, Nottingham LE12 5RD, UK; Centre for Plant Integrative Biology, University of Nottingham, Nottingham LE12 5RD, UK; Division of Plant and Crop Science, School of Biosciences, University of Nottingham, Nottingham LE12 5RD, UK; Centre for Plant Integrative Biology, University of Nottingham, Nottingham LE12 5RD, UK; Division of Plant and Crop Science, School of Biosciences, University of Nottingham, Nottingham LE12 5RD, UK; Centre for Plant Integrative Biology, University of Nottingham, Nottingham LE12 5RD, UK

## Abstract

AUXIN RESISTANCE4 (AXR4) regulates the trafficking of auxin influx carrier AUXIN1 (AUX1), a plasma-membrane protein that predominantly localizes to the endoplasmic reticulum (ER) in the absence of AXR4. In Arabidopsis (*Arabidopsis thaliana*), *AUX1* is a member of a small multigene family comprising 4 highly conserved genes—*AUX1*, *LIKE-AUX1* (*LAX1*), *LAX2*, and *LAX3*. We report here that LAX2 also requires AXR4 for correct localization to the plasma membrane. AXR4 is a plant-specific protein and contains a weakly conserved α/β hydrolase fold domain that is found in several classes of lipid hydrolases and transferases. We have previously proposed that AXR4 may either act as (i) a post-translational modifying enzyme through its α/β hydrolase fold domain or (ii) an ER accessory protein, which is a special class of ER protein that regulates targeting of their cognate partner proteins. Here, we show that AXR4 is unlikely to act as a post-translational modifying enzyme as mutations in several highly conserved amino acids in the α/β hydrolase fold domain can be tolerated and active site residues are missing. We also show that AUX1 and AXR4 physically interact with each other and that AXR4 reduces aggregation of AUX1 in a dose-dependent fashion. Our results suggest that AXR4 acts as an ER accessory protein. A better understanding of AXR4-mediated trafficking of auxin transporters in crop plants will be crucial for improving root traits (designer roots) for better acquisition of water and nutrients for sustainable and resilient agriculture.

## Teaser

AUXIN RESISTANCE4 is an endoplasmic reticulum accessory protein that regulates the trafficking of auxin influx carriers AUXIN1 and *LIKE-AUX2*.

## Introduction

Auxin is a crucial plant hormone that regulates a wide range of biological processes including root, leaf and flower development, apical dominance, vascular development, and tropic responses (Leyser 2006; Vanneste and Friml 2009; Swarup and Bennett 2014; Lavy et al. 2016; Swarup and Bhosale 2019; Truskina et al. 2021). Auxin transport is carrier-mediated and regulated by auxin influx and efflux carriers. AUXIN1/LIKE-AUX1 (AUX1/LAX) family members are the major auxin influx carriers whereas PIN-FORMED (PIN) family and some members of the P-GLYCOPROTEIN/ATP-BINDING CASSETTE B4 (PGP/ABCB) family are major auxin efflux carriers (Swarup and Péret 2012; Swarup and Bennett 2014; Swarup and Bhosale 2019). Auxin is unique amongst plant hormones for exhibiting polar transport. Polarity of auxin movement is established mainly by the asymmetric localization of PIN proteins, which provide intercellular auxin flow in concert with AUX1/LAX proteins (Swarup and Bennett 2014; Swarup and Bhosale 2019).

AUX1/LAX auxin influx carriers are multihelical transmembrane proteins and are localized on the plasma membrane (PM) (Swarup et al. 2001, 2004, 2005; Péret et al. 2012; Swarup and Bhosale 2019). Mutations in *AUX1/LAX* genes result in auxin-regulated developmental defects (reviewed in Swarup and Bhosale 2019). In short, AUX1 plays a major role in root gravitropic responses and root-hair elongation under low phosphate (Bennett et al. 1995; Swarup et al. 2001, 2005; Bhosale et al. 2018; Giri et al. 2018). LAX2 has been shown to regulate vascular patterning in Arabidopsis (*Arabidopsis thaliana*) whereas LAX3 plays a role in lateral root emergence (Swarup et al. 2008; Péret et al. 2012).

We have previously shown that the PM targeting of AUX1 is regulated by AUXIN RESISTANCE 4 (AXR4) (Dharmasiri et al. 2006). *axr4* was initially identified in screens for auxin-resistant root elongation (Hobbie and Estelle 1995) and altered root gravitropism (Simmons et al. 1995). Detailed characterization of the mutant revealed a weak *aux1*-like phenotype (Hobbie and Estelle 1995; Yamamoto and Yamamoto 1998, 1999). AXR4 is an endoplasmic reticulum (ER) protein that contains a single transmembrane helix, and an α/β hydrolase fold domain (Dharmasiri et al. 2006) that is found in several classes of lipid hydrolases and transferases (Mindrebo et al. 2016). Mutations in both *AUX1* and *AXR4* result in a root gravitropic defect (Hobbie and Estelle 1995; Hobbie 2006). Dharmasiri et al. (2006) then revealed that AXR4 regulates PM targeting of AUX1, thus providing the molecular basis of the root gravitropic defect of *axr4*.

In this study, we provide experimental evidence that one other member of the *AUX1/LAX* family, LAX2, requires AXR4 for its correct localization. In addition, using a combination of molecular, genetic and biochemical approaches, we have investigated the mechanistic aspects of AXR4 function. It has been proposed that AXR4 could be an ER accessory protein that regulates the correct folding of AUX1 and prevents it from aggregating in the ER membrane (Dharmasiri et al. 2006). Such accessory proteins are also known as ER-dedicated chaperones or client-specific chaperones that act on a limited number of target proteins, providing assistance with obtaining correct topology, preventing aggregation, and/or promoting ER exit by providing targeting signals for coat protein II loading (Dharwada et al. 2018). Alternatively, AXR4 may act as a post-translational modifying enzyme. AXR4 is a member of the α/β hydrolase family, which includes several classes of proteins such as lipid hydrolases and lipid transferases. Post-translational modifications can influence a protein's localization as well as its activity, turnover, or interaction with other proteins, for example, the addition of mannose-6-phosphate (M6P) residues to soluble acid hydrolases is required for the correct sorting of these protein by M6P receptors (Braulke and Bonifacino 2009).

Our work reveals that AXR4 is unlikely to function as a post-translational modifying enzyme as it is tolerant to single point mutations in several highly conserved amino acids in the α/β hydrolase fold domain and active site residues are not present in a functional orientation. Our topology mapping studies demonstrate that the C-terminal domain of AXR4 resides within the ER lumen. We also show that AXR4 interacts directly with AUX1 in heterologous expression systems, and is required to prevent aggregation of AUX1 thus supporting its role as an ER accessory protein.

## Results

### AXR4 is required for the correct localization of LAX2

**Figure 1.**
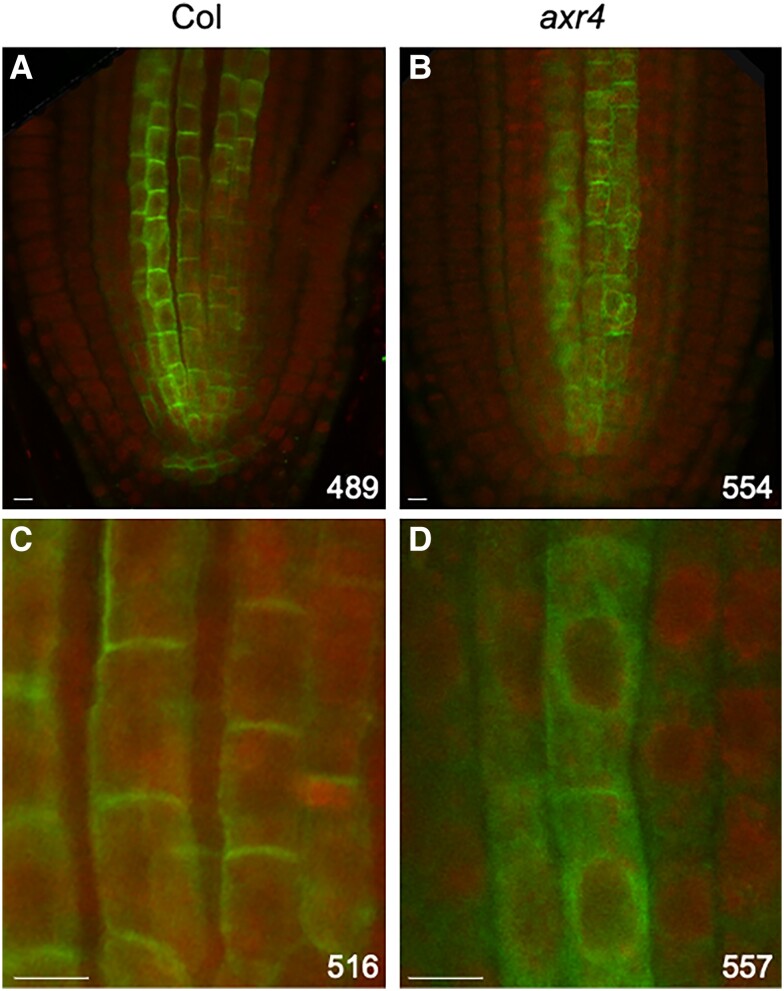
AXR4 regulates the targeting of LAX2. Localization of LAX2 in the roots of Col **A and C)** and *axr4***B and D)** using anti LAX2 primary antibody and Alexa Fluor488 coupled anti-rabbit secondary antibody. Background staining was done using Propidium Iodide. Due to the weaker signal in the *axr4* roots, the *axr4* images **B and D)** were taken at a higher gain and the gain values (volt) are indicated. Scale bar = 5 *µ*M.

Dharmasiri et al. (2006) showed that in the *axr4* mutant AUX1 protein accumulated in the ER instead of being localized correctly to the PM. Here, we tested if AXR4 also regulates the targeting of LAX2. To test this, we used whole mount in situ immunolocalization using anti-LAX2 antibodies (Oh et al. 2020) in the roots of 4 d old wild-type Col-0 or *axr4-2* seedlings. Results show that while the LAX2 signal is very clearly seen on the PM in the wild-type Col-0 seedlings, in the *axr4* mutant background most of the signal appears to be mislocalized inside the cell with only a very weak signal at the PM (Fig. 1). In *axr4* mutants, AUX1 has been shown to be mislocalized in the ER (Dharmasiri et al. 2006). To test if LAX2 also accumulates in the ER in the *axr4* background, in situ co-immunolocalization experiments were done in 4-d old *axr4* seedlings using LAX2 and the ER marker BPL1 antibodies (Dunkley et al. 2006; Oh et al. 2020). Results show that LAX2 colocalizes with the ER marker BPL1 (Supplemental Fig. S1), suggesting that LAX2 is indeed mislocalized to ER in the *axr4* mutant background.

### Mutation in AXR4 phenocopies *lax2*

**Figure 2.**
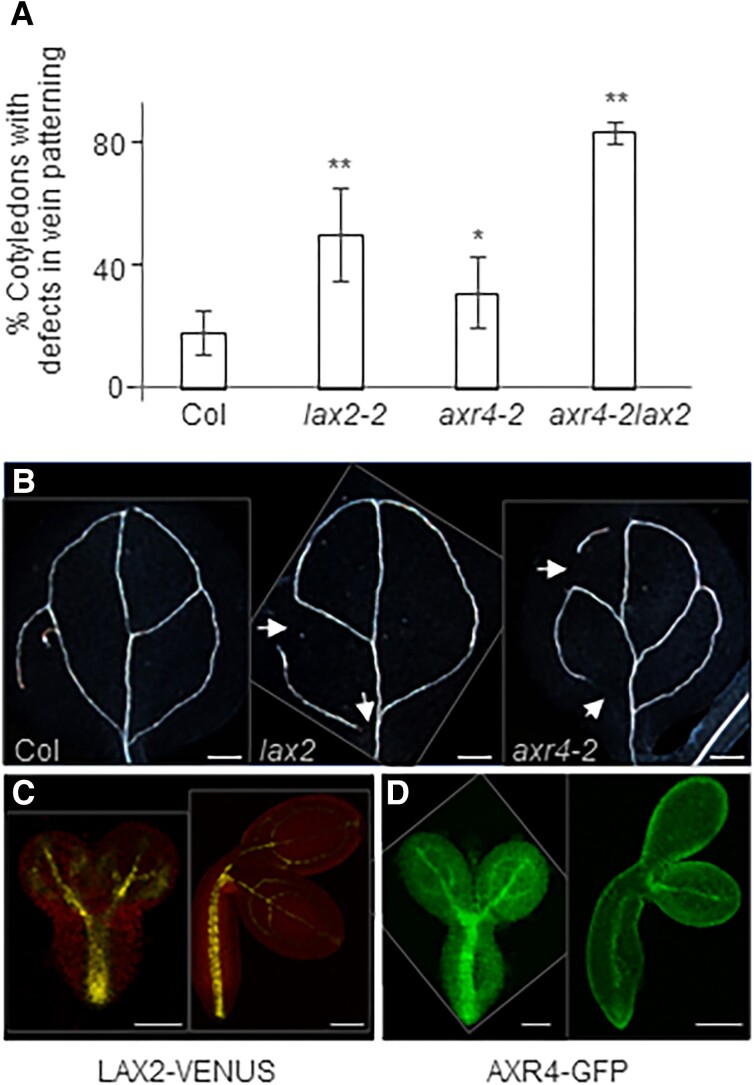
*axr4* mutants phenocopy *lax2*. *axr4* mutants show vascular patterning defect (arrow) in cotyledons **A and B)**. *Indicates significant difference (Student's *t*-test) from wild type Col-0 (**P ≤ 0.01; *P ≤ 0.05). Statistical analysis (Student's *t*-test) also shows that *lax2-2* phenotype is also significantly different from *axr4* (*P* = 0.011624) and *axr4-2lax2* double mutant phenotype is significantly different from *lax2-2* (*P* = 0.000315) and *axr4* (*P* = 3.06E−05). Error bars represent standard deviation. *n* ≥ 50. Both *LAX2* and *AXR4* are expressed during embryo development **C and D)**. Scale bar = 200 *µ*m **B)**; 50 *µ*M **C and D)**.

We next tested the functional implications of mis-targeting of LAX2 in *axr4*. If AXR4 also regulates the targeting of LAX2, the *axr4* mutant should exhibit *lax2-*related defects. *lax2* mutants have been shown to have vascular patterning defects in the cotyledons (Péret et al. 2012). Interestingly, *AXR4* is not expressed in the cotyledons. However, we reasoned that vascular patterning in cotyledons takes place during embryo development, and so we tested the expression of LAX2 and AXR4 during embryo development using *AXR4_Pro_::AXR4-GFP* and *LAX2_Pro_::LAX2-VENUS* reporter lines. We reveal that both *AXR4* and *LAX2* are expressed in the developing embryos (Fig. 2; Supplemental Fig. S2). *AXR4* expression can be detected in globular, transition, heart, late heart, torpedo, and cotyledon stages of embryo development (Fig. 2; Supplemental Fig. S2). *AXR4* is weakly expressed in almost all cell files in the embryo but is more pronounced in the hypophysis by the late heart stage, and from the early cotyledon stage onward, the expression becomes more localized in the vascular tissues (Fig. 2; Supplemental Fig. S2). In comparison, during the early stages of embryo development, *LAX2* expression is more pronounced in the hypophysis and the central basal parts of the embryo but weak expression can still be seen in the upper central part of the embryo and may be a very early marker for vascular development. The expression becomes more pronounced in the vascular tissues in later stages of embryo development (Supplemental Fig. S2).

Once it became clear that *AXR4* is co-expressed with *LAX2* during embryo development, we then tested if *axr4* mutants show any vascular patterning defect in the cotyledons as reported for *lax2* (Péret et al. 2012). Five-day old Col-0, *lax2*, *axr4*, and *axr4lax2* double mutant seedlings were cleared and vascular patterning studied. We reveal that *lax2*, *axr4,* and *axr4lax2* all show higher propensity of discontinuity in vascular strands compared with wild type Col-0 (Fig. 2, A and B). Interestingly, *axr4lax2* showed a more severe vascular patterning defect compared with the single mutants but *axr4* defect is less pronounced than *lax2* (Fig. 2A). Therefore, we conclude that *axr4* has a weak *lax2* phenotype (Fig. 2, A and B).

### Topology mapping studies suggest AXR4 C-terminal domain resides within the ER lumen

*AtAXR4* encodes a 473 amino acid ER protein with a predicted molecular mass of 52.4 kDa (Dharmasiri et al. 2006). Aramemnon, a plant membrane protein database (Schwacke and Flügge 2018), was used to generate topology and transmembrane (TM) domain prediction by defining a consensus of 18 programs such as TopPredII (Claros and von Heijne 1994), TmHMM (Sonnhammer et al. 1998) and DAS membrane prediction server (Cserzö et al. 1997). The majority of the predictions, such as Cserzo et al. (2004), suggest that AXR4 is an integral membrane protein with one transmembrane domain located near the N-terminus, spanning between amino acid residues 46 and 70 (Supplemental Fig. S3). However, other TM prediction programs, such as TmPred, Scampi (Bernsel et al. 2008), and PHDhtm (Combet et al. 2000), suggest that AXR4 has 2 to 3 TMs (Supplemental Fig. S3). To investigate this further, we performed topology mapping by protease-protection experiments on wildtype Columbia microsomes probed with an anti-AXR4 antibody. This antibody is specific to AXR4 as revealed by in situ immunolocalization and a single correct size band on western blots (Supplemental Fig. S4).

In a protease-protection experiment, any part of the protein inside the intact microsomes would be protected against protease digestion (Gottlieb et al. 2012; Besingi and Clark 2015). Microsomes were extracted from Arabidopsis root cultures and were treated with Proteinase K in the presence or absence of Triton X-100 (a nonionic detergent used for membrane permeabilization). Samples were taken at 2, 10, 30, and 60 min post-treatment and analyzed by western immunodetection using anti-AXR4 antibodies. Results show that a 55 kDa band is clearly detected in intact microsome preparations (− Triton X-100) 2 min post-treatment and the size of the band diminishes over the time course (Supplemental Fig. S5A). In contrast, in samples where microsomal membrane integrity is compromised (+ Triton X-100), no band is detected upon Proteinase K treatment (Supplemental Fig. S5B). These results suggest that most of AXR4 resides inside the ER lumen and hence is protected from Proteinase K digestion whereas a small part protrudes out of the ER membrane and is exposed to Proteinase K and degraded. Taken together with the TM prediction studies, we conclude that AXR4 has one transmembrane domain located near the N-terminus, spanning between amino acids 46 and 70 with the small N terminus protruding outside the ER into the cytosol and the large carboxyl-terminal residing inside the ER lumen.

### AXR4 contains a weakly conserved αβ hydrolase domain that is flexible to point mutations and appears not to play a role in AXR4 function

**Figure 3.**
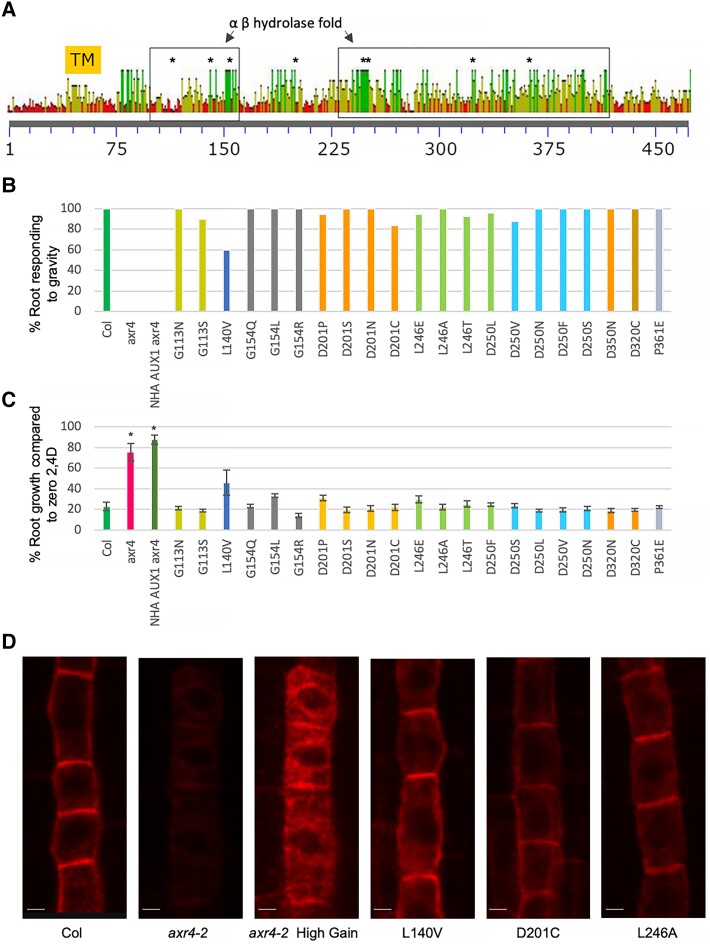
Single point mutations in AXR4 α/β hydrolase fold domain can rescue *axr4* root growth defect. Multiple sequence alignment of plant AXR4-like sequences showing consensus transmembrane region (TM) and α/β hydrolase fold domain **A**). Numbers in A refer to the amino acids. Point mutations were generated in *AtAXR4* gene and were then introduced in *axr4* mutant background. The IVM lines were named based on the amino acid substitutions. Homozygous T3 lines were then analyzed for root gravitropism **B**) and sensitivity to 50 nM 2,4D **C**). Error bars represent standard error. *Indicates significant difference from Col-0 control (*P*≤0.01). In situ immunolocalization of HA-AUX in controls and a few IVM lines (L140V, D201C, and L246A) using anti-HA primary antibody and Alexa Fluor 543 coupled secondary antibody **D**). Scale bar = 2 *µ*M.

AXR4 may act as a post-translational modifying enzyme, as the bioinformatics studies suggest that AXR4 contains a weakly conserved α/β hydrolase fold domain (esterase lipase domain) that is found in several classes of lipid hydrolases and transferases (Mindrebo et al. 2016). To investigate the role of the α/β hydrolase fold in AXR4 function, highly conserved amino acids within α/β hydrolase fold domains were identified using over 100 interkingdom sequences containing this domain (Fig. 3A; Supplemental Fig. S6). Out of the amino acids that were conserved between plant AXR4-like sequences, 18 amino acids appear to be highly conserved throughout all sequences. The 7 most highly conserved amino acids and one amino acid as a control (G118) were selected for site-directed mutagenesis to probe their role in AXR4 function (Fig. 3A; Supplemental Fig. S6). One of these amino acids, D250, might occur in the catalytic triad SHD, which has been shown to be essential for α/β hydrolase enzyme activity (Rauwerdink and Kazlauskas 2015).

A PCR-based approach was used for site-directed mutagenesis to create random mutations at the chosen target sites, allowing a single amino acid to be replaced with up to 16 different amino acid combinations. Mutated *mAXR4*-*GFP* transgene constructs were transformed into *_axr4-2_NHA-AUX1* background. Several transgenic lines were isolated and amino acid substitutions were confirmed by sequencing that identified 34 different amino acid substitutions at 8 chosen targets (Table 1). The substitutions ranged from very subtle changes such as Leu246Ala to some drastic changes such as Asp320Arg.

**Table 1.** Summary of point mutations in AXR4

Position	Amino acid change
113	Gly → Asn; Ser
140	Leu → Gly; Val
154	Gly → Arg; Gln; Glu; Leu; Lys
201	Asp → Asn; Cys; Pro; Ser
246	Leu → Ala; Glu; Thr
250	Asp → Ala; Asn; His; Gly; Leu; Phe; Ser; Tyr; Val
320	Asp → Arg; Asn; Cys; Ile; Ser
361	Pro → Arg; Glu; Leu; Trp

To investigate the effects of these changes on AXR4 function, homozygous T3 *_axr4-2_mAXR4-GFP* lines were then used to test if they could restore the mutant defect of *axr4-2*. *axr4* mutant roots are agravitropic and are resistant to the inhibitory concentrations of synthetic auxin 2,4-D (Dharmasiri et al. 2006). We first tested the root gravitropic response of these *_axr4-2_mAXR4-GFP* lines using root bending assays (Swarup et al. 2004; Muller et al. 2018). Seedlings were grown for 4 d vertically, then the plates were turned at 90° and the bending response of the roots was scored. Results show that within 6 h all wildtype Col-0 seedlings responded to the gravity stimulus while *axr4* mutant seedlings did not respond to the gravity stimulus even after 10 h of the gravity stimulus. In comparison, all *mAXR4-GFP* lines responded to the gravity stimulus to varying degrees and most lines show a high number (>80%) of seedlings responding to the gravity vector within 10 h of the gravity stimulus except *mAXR4-GFP L140V* line where only 60% seedlings responded to the gravity stimulus (Fig. 3B; Supplemental Fig. S7B).

We next tested the root growth response of these *_axr4-2_mAXR4-GFP* lines on inhibitory concentrations of 2,4D. Results revealed that root growth of wildtype Col-0 seedlings is severely inhibited at 50 and 100 nM 2,4D but *axr4* and *_axr4-2_NHA-AUX1* roots are resistant to these inhibitory concentrations of 2,4D (Fig. 3C; Supplemental Fig. S7A). In comparison, root growth of all *_axr4-2_mAXR4-GFP* lines shows high sensitivity to 2,4D and most of these lines show a growth response very similar to wildtype Col-0 controls (Supplemental Fig. S7A). One line (L140V) showed a root growth inhibition very similar to wildtype at 100 nM 2,4D but L140V roots were slightly less sensitive to inhibition at 50 nM 2,4D, suggesting it is only a partial rescue (Fig. 3C; Supplemental Fig. S7A).

It is evident that at the phenotypic level, all *_axr4_mAXR4-GFP* lines carrying point mutations in *AXR4* transgene can essentially fully complement the *axr4* mutant defect. To investigate the molecular basis of this rescue, we next performed in situ immunolocalization to test HA-AUX1 PM localization in these lines. In contrast to *axr4* mutants where AUX1 PM targeting is compromised, all the *mAXR4-GFP* transgenic lines showed clear PM targeting of HA-AUX1 (Fig. 3D). Interestingly, line L140V only showed a partial rescue, and also showed a clear PM localization of AUX1 (Fig. 3D). As AXR4 also regulates trafficking of LAX2, we next tested the localization of LAX2 in these lines. Similar to the results obtained for HA-AUX1 localization, in situ immunolocalization results show that the LAX2 targeting defect in *axr4* is also restored in these *_axr4_mAXR4-GFP* lines (Supplemental Fig. S8). These results suggest that AXR4 can tolerate point mutations within the α/β hydrolase fold domain, with all amino acid changes resulting in a functional AXR4 protein that can restore AXR4 function.

To investigate this further, we used the protein 3D-structure prediction program Phyre2 (Kelley et al. 2015). For AXR4, 117 of the 120 retrieved structures had alpha-beta hydrolase folds with a confidence level of 99% or higher. The remaining 3 were not ranked high enough for homology models to be made. Hydrolases have a common active site mechanism, involving the so-called catalytic triad (Rauwerdink and Kazlauskas 2015), whose position in the 3D structure must be conserved to retain catalytic activity. AXR4 does not conserve these residues in the expected positions and a visual examination for the presumptive active site confirms that residues are in the wrong 3D orientation (Supplemental Fig. S9). These results also strongly suggest that AXR4 cannot function as an α/β hydrolase.

### AXR4 interacts directly with AUX1 and prevents AUX1 aggregation in a dose-dependent fashion and thus may function as an ER accessory protein

**Figure 4.**
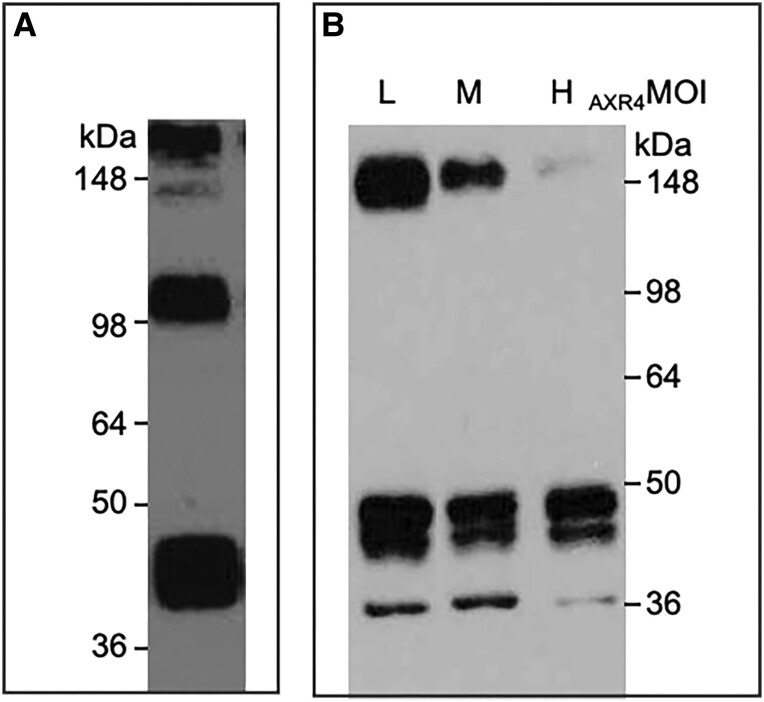
AXR4 can reduce AUX1 aggregate formation in a dose-dependent fashion. AUX1 has high propensity to form multimeric aggregate in insect cells **A**). AXR4 reduces AUX1 aggregate formation in a dose-dependent manner **B**). AUX1 was expressed alone **A)** or co-expressed with AXR4 **B)**, with low (L; 0.1), medium (M; 1), or high (H; 10) MOI.

ER accessory proteins such as Super high Histidine Resistant3 (Shr3p), Receptor Associated Protein (RAP), Chitin Synthase related7 (Chs7), Phosphate Metabolism86 (Pho86), and Glucose Signaling Factor2 (Gsf2) are necessary for the correct localization of their targets General Amino acid Permease1, Lipoprotein Receptor-related Protein, Chitin Synthase3 (Chs3), Phosphate Metabolism84 and HEXOSE TRANSPORTER1, respectively, and in their respective mutants these specific targets aggregate within the ER (Ljungdahl et al. 1992; Bu et al. 1995; Herrmann et al. 1999; Trilla et al. 1999; Kota et al. 2007). To test whether AXR4 has a similar function, we co-expressed _HA-HIS_AXR4 and _HIS-FLAG_AUX1 in insect cells using a baculovirus expression system. Expression of _His-FLAG_AUX1 alone resulted in detection of a monomeric protein band at ∼45 kDa, as well as higher molecular weight species of ∼100 and >150 kDa (Fig. 4A), presumably representing SDS-resistant aggregation states of AUX1. Co-expression of both AUX1 and AXR4 baculovirus constructs was then employed, varying the multiplicity of infection (MOI) to give the desired low, medium or high expression of AXR4. The results from the co-expression studies showed that the presence of AXR4 reduced the intensity of higher order bands for AUX1 when compared with expression without AXR4 (Fig. 4B). We conclude that AUX1 has a propensity to form aggregates and AXR4 can reduce AUX1 aggregate formation in a dose-dependent manner when co-expressed together (Fig. 4).

**Figure 5.**
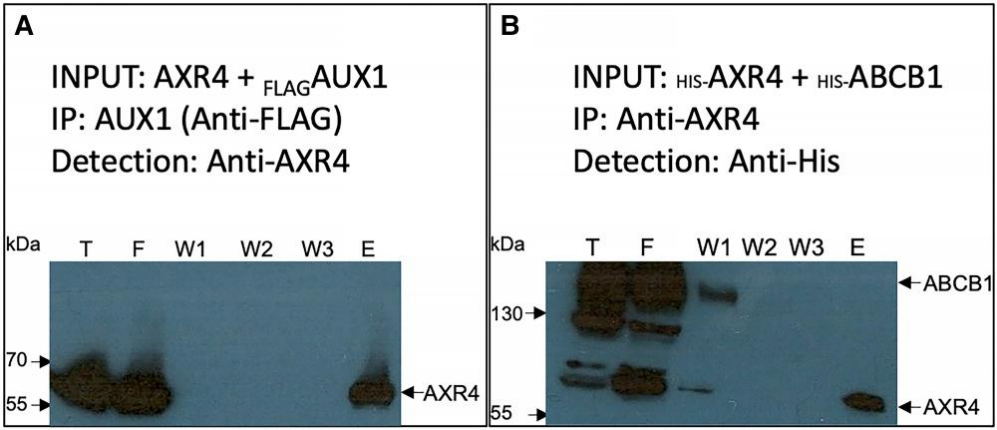
AXR4 and AUX1 interact with each other. AXR4 and _FLAG_AUX1 were co-expressed in insect cells and _FLAG_AUX1 immunoprecipitated using anti-FLAG resin. AXR4 can be detected in the pull down by western immunodetection using anti-AXR4 antibodies **A**). As control, _HIS-_AXR4 and _HIS-_ABCB1 were co-expressed in insect cells and _HIS-_AXR4 immunoprecipitated using anti-AXR4 antibodies. ABCB1 cannot be detected in the pull downs by western immunodetection using anti-HIS antibodies **B**). T, total extract; F, flowthrough; W, wash; E, elution.

Evidence has also shown that ER accessory proteins interact directly with their targets, for example Kota et al. (2007) have shown a direct interaction between Shr3p and its targets. Therefore, co-immunoprecipitation (Co-IP) experiments in heterologous systems were used to see if the interaction between AXR4 and AUX1 is direct. Co-IP experiments were performed on baculovirus cell lysates expressing AUX1 and AXR4. AXR4 can be immunodetected in _FLAG_AUX1 pull downs when co-expressed with AUX1 (Fig. 5A) but not when expressed alone (Supplemental Fig. S10) suggesting an interaction between AUX1 and AXR4. To rule out the possibility that the interaction seen was not simply due to overexpression of 2 highly expressed proteins, a control Co-IP experiment was designed where _His_AXR4 was co-expressed with the mammalian multidrug pump _His_ABCB1 (Mittra et al. 2017). The rationale for using the same epitope tag (His) for both AXR4 and ABCB1 was that the 2 proteins could be distinguished based on their size differences: AXR4 being 55 kDa and ABCB1 130 kDa. We show that despite both AXR4 and ABCB1 being highly expressed in insect cells, ABCB1 cannot be pulled down with anti-AXR4 (Fig. 5B) ruling out the possibility that the interaction between AUX1 and AXR4 is just an artifact due to high expression levels. In summary, Co-IP experiments have detected a specific interaction between AXR4 and AUX1, and a possible mode of action consistent with AXR4 functioning as an ER accessory protein.

## Discussion

We have previously shown that AXR4 regulates PM targeting of AUX1 (Dharmasiri et al. 2006). We now have provided evidence that AXR4 is required for the correct localization of another member of the AUX1/LAX family, LAX2. Like AUX1, LAX2 also predominantly accumulates in the ER in *axr4* mutants. Defects in AUX1 and LAX2 mislocalization in *axr4* are also manifested at phenotypic levels, as *axr4* mutants show weak *aux1* (root gravitropic defect) and *lax2* (vascular patterning defects in the cotyledons) phenotypes. Our in situ localization experiments show weak LAX2 (and AUX1) signals at the PM in *axr4* mutants. It is not clear why there is a weak LAX2 and AUX1 signal at the PM. Our interpretation is that most AUX1/LAX2 proteins are unable to fold correctly in the *axr4* mutants and aggregate in the ER. However, a small fraction that can fold correctly by chance leave the ER and can get to the PM. This also explains the weak *lax2* and *aux1* phenotype of *axr4* mutants. Interestingly, for the vascular patterning defect, though we see a weaker *axr4* phenotype compared with *lax2*, *axr4lax2* double mutants show a more severe vascular patterning defect compared with the single mutants (Fig. 2A). This is not surprising if we consider that *AUX1* is also expressed in the vascular tissues during embryo development (Robert et al. 2015). As AXR4 regulates the trafficking of both AUX1 and LAX2, this could explain the more severe vascular patterning defect of *axr4lax2*.

Despite high similarity at the protein level and their dependence on AXR4, AUX1, and LAX2 proteins appear to be under distinct trafficking regulation. Péret et al. (2012) had previously shown that when expressed under *AUX1* promoter, LAX2 was unable to rescue *aux1* mutant defect. Localization studies revealed that LAX2 was mislocalized in cells where it is not normally expressed but was correctly localized in its native expression domain. Domain swap experiments between AUX1 and LAX2 showed that the N-terminal half of AUX1 is required for the correct localization of AUX1/LAX2 chimeric fusion protein in *AUX1* expressing cells (Péret et al. 2012). Further studies will be required to identify additional trafficking factors required for the correct targeting of these PM proteins.

How AXR4 facilitates the correct targeting of AUX1/LAX2 proteins to the PM is unclear. The large C terminal domain of AXR4 contains highly conserved regions with high amino acid similarity when aligned with AXR4-like homologs from other species, therefore, this domain may be important for its function, possibly interacting with other proteins. Our topology mapping studies show that AXR4 is a single transmembrane protein. In western blot analysis, the AXR4 protein migrates on the SDS-PAGE at 55 kDa which is close to the predicted molecular weight of 53 kDa for AXR4. Using Proteinase K digestion of intact microsomes we have shown the large C-terminal domain of AXR4 was protected from the proteolytic activity and requires Triton X-100 to permeabilize the microsomes before the large C terminal part of AXR4 is degraded. This indicates that the large carboxyl terminal hydrophilic portion of the protein protrudes into the ER lumen, while the short N-terminus is exposed to the cytoplasmic side.

The large C terminal part of AXR4 also contains a weakly conserved α/β hydrolase fold domain (esterase lipase domain). The α/β hydrolase fold family is one of the most versatile and widespread protein folds known, and over 50 structures have been solved, including proteases, lipases, esterases, dehalogenases, peroxidases, and epoxide hydrolases (Nardini and Dijkstra 1999; Holmquist 2000; Marchler-Bauer et al. 2007; Mindrebo et al. 2016). In this study, 117 proteins known to have this fold were retrieved. The common structure of the α/β hydrolase fold domain shared by the members of this family, suggests that despite the different functions, these proteins share common mechanisms of protein folding and processing, and have been shown to be involved in trafficking (De Jaco et al. 2010).

Our studies suggest that AXR4 is not a post-translational modifying enzyme as mutations in 8 highly conserved amino acids in the α/β hydrolase fold domain are tolerated. These amino acids were identified based on highly conserved regions between AXR4 homologs in other plant species, and interkingdom genes containing the α/β hydrolase fold domain. In the 2,4-D screen, the majority of these lines carrying single point mutations in these highly conserved amino acids rescue the *axr4* mutant defect, with one line L140V showing a partial rescue. This is also consistent with the gravitropism assay, where all mutants respond to gravity within 10 h, while *axr4* itself does not respond within this time scale. To further confirm that AXR4 function was restored in the mutant lines, in situ immunolocalization of LAX2 and NHA-AUX1 was used to see whether they were correctly targeted to the PM. These results confirm that LAX2 and AUX1 are correctly targeted within the mutants, while aggregating within the ER in *axr4*, suggesting that all amino acid changes lead to a functional AXR4 protein irrespective of the severity of amino acid substitutions. It is possible that the amino acid change has only a relatively small change on the structure and, therefore, only slightly reduces AXR4 efficiency.

Taken together, our results suggest that it is unlikely that AXR4 is a post-translational modifying enzyme, as it would be more sensitive to amino acid changes if it had an enzymatic function. This is also supported by our modeling studies. At molecular level, the α/β hydrolase fold consists of 8 β sheets and 6 α helices in a characteristic arrangement. Hydrolases have a common active site mechanism, involving the so-called catalytic triad (Rauwerdink and Kazlauskas 2015). This involves 3 residues that come together in different folds and are arranged in 3D in the order: acid (often D), base (often H but can be K or R), and nucleophile (S, T, or C) which actually hydrolyzes the substrate (Holmquist 2000; Marchler-Bauer et al. 2007; Mindrebo et al. 2016).

The hydrolases to which AXR4 fits well have the most common triad, namely D-H-S (Supplemental Fig. S9). However, in AXR4, 2 of the equivalent positions do not have residues capable of producing a functional triad (Supplemental Fig. S9). D250 should be a Serine residue, so it is not surprising that mutations at this position are tolerated. The inability to identify a single amino acid substitution that results in loss of function makes AXR4 an interesting protein for structural studies. This may also explain why missense alleles of *axr4* have not been identified in numerous 2,4-D screens. The only mutations discovered for *AXR4* are insertions (T-DNA and γ-radiated) and those EMS mutants that result in stop codons. The lack of missense mutations in *AXR4* that cause loss of function, could be because AXR4 has flexibility within its structure and can cope with single amino acid changes without losing function.

**Figure 6.**
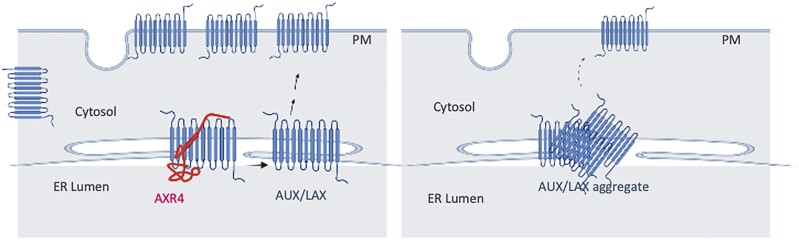
Model for AXR4 function. AXR4 facilitates folding of AUX1 and LAX2 proteins, and functional AUX1 and LAX2 proteins are trafficked to the PM **A**). In the absence of AXR4, AUX1, and LAX2 proteins that cannot fold correctly form multimeric aggregates and are retained in the ER **B**).

While AXR4 does not seem to function as a post-translational modifying enzyme through the α/β hydrolase fold domain, our experiments support that AXR4 functions as an ER accessory protein (Fig. 6). This is based on the observation that AXR4 directly interacts with AUX1 and reduces aggregation of AUX1 in a dose-dependent manner. ER accessory proteins have been shown to be involved in providing correct folding and preventing aggregation. For example, the loss of PHOSPHATE TRANSPORTER TRAFFIC FACILITATOR1 (PHF1) in Arabidopsis leads to an abnormal accumulation of its target protein PHOSPHATE TRANSPORTER1 (PHT1) within the ER, and loss of correct localization to the PM (González et al. 2005). Studies on other ER accessory proteins have shown that this abnormal accumulation is the result of aggregation. The mammalian LOW-DENSITY LIPOPROTEIN (LDL) receptor family aggregates in the ER in the absence of its ER accessory protein RAP (Bu et al. 1995; Bu and Schwartz 1998).

There has been a lot of work on ER accessory proteins showing physical interaction with their direct targets. Previous work has shown that the ER accessory protein TANGO1 directly interacts with its cognate target collagen VII through immunoprecipitation (Saito et al. 2009). Our data from the Co-IP studies of AXR4 and AUX1 provides strong evidence that these 2 proteins interact, adding weight to a reported yeast-2-hybrid interaction (Altmann et al. 2020). The interaction of AXR4 with AUX1 is consistent with the proposed role as an ER accessory protein, functioning as a molecular chaperone providing correct structure and/or reducing aggregation (Dharmasiri et al. 2006).

ER accessory proteins often have a single target or are involved in the correct targeting of a whole family or subset of proteins. Chs7 for example has been shown to be specific to a single protein (Chs3) trafficking out of the ER (Trilla et al. 1999), while RAP is involved in the specific trafficking of the LDL receptor family in humans (*Homo sapiens*) (Bu et al. 1995), and Shr3p is required for the correct targeting of the amino acid permease family (18 members) within yeast (*Saccharomyces cerevisiae*) (Gilstring et al. 1999; Kota and Ljungdahl 2005). Members of the AUX1/LAX family show high similarity with each other (76% to 82%) and we have provided evidence that AXR4 regulates the targeting of AUX1 and LAX2 and it is tempting to speculate that AXR4 may act as an ER accessory protein for the whole family.

Currently, there is little evidence on the mechanism of these ER accessory proteins, and further research is required to determine how they achieve correct protein folding and exit of their target from the ER. It is likely that is more than one mechanism as some ER accessory proteins stay within the ER (Shr3 and Pho86) (Kuehn et al. 1996; Lau et al. 2000), whereas others such as Chs7 and Gsf2 are exported from the ER with their substrates and have a cytosolic KXKXX signal to allow for COPI-mediated recycling back to the ER (Sherwood and Carlson 1999; Dharwada et al. 2018). We analyzed AXR4 to further understand its role as an ER accessory protein and noted a potential diacidic motif (DSD) is present on the N-terminal cytosolic domain of AXR4 (Supplemental Fig. S11). This motif (D/E)X(D/E) has been shown to be involved in the export of transmembrane proteins from the ER in plants as well as other systems (Hanton et al. 2005). In addition, a canonical dilysine motif KNKPK is also present within the N-terminal cytosolic domain of AXR4 (Supplemental Fig. S11). This motif KXKXX has been shown to interact with COPI vesicles and to be involved in ER retention in plants (reviewed by Gao et al. 2014). It is therefore possible that AXR4 plays a role in correct folding of the AUX/LAX family and a direct role in their transport out of the ER.

## Materials and methods

### Plant materials and growth conditions

The Arabidopsis (*A. thaliana*) *axr4-2*, *aux1-22*, *lax1*, and *lax2* mutants have been described previously (Swarup et al. 2004, 2008; Dharmasiri et al. 2006; Péret et al. 2012). Plants were grown on vertical MS (2.15 g/L, 1% [w/v] bacto agar) plates at 23°C, under continuous light at 150 *µ*mol m^−2^ s^−1^. Gravitropic assays were performed as previously described (Swarup et al. 2004, 2005). Root elongation assays were performed as previously described (Dharmasiri et al. 2006; Péret et al. 2012). Primary root length was measured using the NeuronJ plugin for the ImageJ software (*n* > 20 per line). For vein patterning in cotyledons, 5-d old seedlings were cleared overnight at room temperature using chloral hydrate solution (chloral hydrate, glycerol, water 8:1:2 w/v/v) and vein patterning was observed using a Zeiss light microscope (*n* > 50 per line).

### Isolation of Arabidopsis microsomes

A microsomal membrane fraction was prepared using Arabidopsis root cultures grown in Gamborg B5 medium (0.32% [w/v] for 4- to 5-wk in the dark, 100 rpm shaking at 20°C to 22°C). Five grams of root tissue were homogenized in homogenization buffer (0.5 M sucrose, 50 mM HEPES-OH, pH 7.5, 0.5% [w/v] polyvinyl polypyrrolidone, 0.1% [w/v] sodium ascorbate, 1.0 mM DTT, and Complete Protease Inhibitor Cocktail [Sigma-Aldrich]) and filtered through 100 *μ*M nylon mesh. The filtrate was centrifuged for 12 min at 2,800 × *g* at 4°C, and the microsomal membrane fraction was pelleted further by centrifugation at 100,000 × *g* for 1 h at 4°C. The microsomal pellet was resuspended in a PBS solubilization buffer.

### Topology mapping

Columbia microsomes were resuspended in fXa buffer (250 mM sorbitol, 100 mM NaCl, 20 mM Tris–HCl, pH 7.5, 1 mM EDTA). Proteinase K was added to the microsomes in the presence or absence of 0.2% [v/v] Triton X-100, and incubated at 37°C for 2, 10, 30, and 60 min.

### Baculovirus expression experiments

N-His_6_3xFLAG-AUX1, L2-His_6_3xFLAG-AUX1, and His_6_HA-AXR4 have been described previously (Carrier et al. 2009). Recombinant AXR4 bacmid DNA was constructed using the Bac-2-Bac system (Invitrogen), following manufacturer's instructions, with a HA-His tag (Y P Y D V P D Y A H H H H H H) introduced to the C terminus. After PCR screening of the basmid DNA to ensure the correct insertion of *AXR4* cDNA, recombinant virus was generated by Cellfectin-mediated transfection of Sf9 cell monolayers to generate viruses for the recombinant protein experiments.

For all baculovirus expression experiments, the Sf9 insect cell line (Vaughn et al. 1977) derived from ovarian tissue of *Spodoptera frugiperda* was used. All the insect cell manipulations were performed using standard cell culture techniques and grown as previously described (Carrier et al. 2009). For baculovirus infection, Sf9 cells were seeded in 10 mL aliquots at a cell density of 1 × 10^6^ cells and left to grow until cell density of 2 × 10^6^ cells was reached. Aliquots of virus inoculums were added to achieve a MOI of ∼0.1, 1, and 10. The cells were incubated at 28°C for 48 h before being harvested by centrifugation (500 × *g*, 5 min at 4°C), and resuspended in PBS or IP lysis/wash buffer (0.025 M Tris, 0.15 M NaCl, 0.001 M EDTA, 1% [v/v] NP-40, 5% [v/v] glycerol; pH 7.4) with protease inhibitors, and sonicated twice to lyse cells. Western blots were performed to confirm the expression of tagged AUX1 and AXR4 protein. Twenty micrograms of the cell lysate were loaded and separated on 15% SDS-PAGE gel followed by blotting onto a nitrocellulose membrane. Western detection of proteins was performed using anti-FLAG (1:2,000 dilution), anti-His (1:1,000 dilution), anti-HA (1:1,000 dilution), and anti-AXR4 (1:10,000 dilution) antibodies (previously described by Oh et al. 2020). Horseradish peroxidase coupled secondary anti-Mouse or anti-Sheep antibodies (Invitrogen) were used at a dilution of 1:5,000, and enhanced chemiluminescence (SuperSignal West Pico, Pierce) was used for detection.

### Affinity purification and Co-IP

For the Co-IP experiments, the Pierce Co-IP Kit (Thermo Scientific) was used according to manufacturer's instructions. Anti-AXR4 and Anti-Flag were used at 75 and 40 *μ*g/μL respectively, with 50 *μ*L resin. One hundred and fifty microliters of sample in IP lysis/wash buffer was added to each column for each pull-down experiment, and binding occurred overnight at 4°C. Columns were washed 3 times with IP lysis/wash buffer, and then the precipitate was eluted with Elution Buffer (pH 2.8, containing primary amine).

### In vitro mutagenesis

A 3 step PCR approach was used to generate the site-directed mutations (Supplemental Table S1). The primers for this site-directed mutagenesis had the first 2 bases of the amino acid of interest substituted by “N” allowing up to 16 different amino acid changes at a single amino acid position. The PCR product was then cloned into *pENTR11 AXR4:AXR4-GFP* vector produced previously by Dharmasiri et al. (2006), using internal restriction site BamHI and Asp718 replacing the wildtype gene. The clones were sequenced and the mutated *AXR4-GFP* entry vectors were then recombined into Gateway destination vector pGWB7 and transformed into *Agrobacterium tumefaciens* (C58) and Arabidopsis *_axr4-2_NHA AUX1* background and selected on hygromycin plates using routine protocols as described previously (Bhosale et al. 2018).

### Immunolocalization

Four-day-old seedlings were fixed and immunolocalization experiments were performed as described previously (Oh et al. 2020) and visualized using confocal microscopy.

### Histochemical GUS staining

GUS staining was done as described previously (Péret et al. 2012). Plants were cleared for 24 h in 1 M chloral hydrate and 33% [v/v] glycerol. Seedlings were mounted in 50% [v/v] glycerol and observed with a Leica DMRB microscope.

### Protein structure prediction/modeling

The PHYRE 2 server (Kelley et al. 2015) was used for structure prediction because it returns multiple model structures ranked by a combination of confidence and percentage amino acid identity, and includes the intermediate analysis data, pairwise sequence/structure alignments. The AXR4 protein sequence was submitted to it using the normal mode, and the complete output is in Supplemental Data Set 1. The data can be accessed by unzipping the file and then opening in a web browser the file entitled summary.html. Data on active site residues in known structures have been obtained from the Catalytic Site Atlas (Furnham et al. 2014) by PHYRE2 itself and depicted in pairwise alignments by red rectangles. Model and known 3D structures have been visualized and analyzed using UCSF ChimeraX (Pettersen et al. 2021).

### Statistical analysis

Two main statistical methods were used in data analysis; descriptive statistics, which summarizes the data using mean and standard deviation, and inferential statistics, which uses the Student's *t*-test (2-tailed distribution, 2 sample unequal variance).

### Accession numbers

Sequence data from this article can be found in the GenBank/EMBL data libraries under the following accession numbers: At1g54990 (*AXR4*), At2g38120 (*AUX1*), At5g01240 (*LAX1*), At2g21050 (*LAX2*), and At1g77690 (*LAX3*).

## Supplementary Material

kiad506_Supplementary_Data

## Data Availability

The data underlying this article are available in the article and in its online supplementary material.

## Supplemental data

The following materials are available in the online version of this article.

**Supplemental Figure S1.** LAX2 colocalizes with ER marker BPL1 in *axr4* root cells as revealed by in situ immunolocalization.

**Supplemental Figure S2.** *AXR4* is expressed in the expression domains of *LAX2* in the developing embryos.

**Supplemental Figure S3.** Topology mapping studies created by Aramemnon (Schwacke and Flügge 2018) strongly suggest that AXR4 has one transmembrane domain.

**Supplemental Figure S4.** AXR4 antibodies are specific for AXR4.

**Supplemental Figure S5.** Protease-protection experiment.

**Supplemental Figure S6.** Graphic representation of AtAXR4 showing highly conserved residues from the multisequence alignment of AXR4-like sequences.

**Supplemental Figure S7.** Single-point mutations in AXR4 are tolerated.

**Supplemental Figure S8.** In situ immunolocalization of LAX2 in Arabidopsis roots in controls (Columbia, *axr4*) and a few IVM lines (G113S, L140V, D201C, L246A, and D250V).

**Supplemental Figure S9.** The catalytic site is inactive in AXR4.

**Supplemental Figure S10.** Control pull-down experiments show that AXR4 can NOT be detected in the pull down with anti-FLAG resin when AXR4 was expressed alone.

**Supplemental Figure S11.** Motif analysis for AXR4.

**Supplemental Table S1.** Primers used for random site-directed mutagenesis in the conserved amino acids in the α/β hydrolase fold domain of AXR4.

**Supplemental Table S2.** Primers used for genotyping IVM lines.

**Supplemental Data Set 1.** PHYRE2 output for AXR4.

## Dive Curated Terms

The following phenotypic, genotypic, and functional terms are of significance to the work described in this paper:

axr4 Gramene: AT1G54990
axr4 Araport: AT1G54990

AUX1 Gramene: AT2G38120
AUX1 Araport: AT2G38120

ABCB1 Gramene: AT2G36910
ABCB1 Araport: AT2G36910

LAX3 Gramene: AT1G77690
LAX3 Araport: AT1G77690

SHD Gramene: AT4G24190
SHD Araport: AT4G24190
