# Supplementary material for: Mechanistic insight into the role of AUXIN RESISTANCE4 in trafficking of AUXIN1 and LIKE AUX1-2
Source: Plant Physiol. 2023 Sep 30;194(1):422–33. doi: 10.1093/plphys/kiad506 (PMC10756756; doi:10.1093/plphys/kiad506)
Supplement: kiad506_Supplementary_Data [file kiad506_supplementary_data.zip › Suppemental Dataset S1/5445e4f6eaa2385e-AtAXR4/aligs/c2hdwB_.70.alig.html]

Phyre 2 alignment of AtAXR4\_\_\_ with c2hdwB\_


|  |  |
| --- | --- |
| Return to main results | Retrieve Phyre Job Id |

|  |  |  |  |  |  |  |  |  |  |  |  |  |  |  |  |  |  |  |  |  |  |  |  |  |  |
| --- | --- | --- | --- | --- | --- | --- | --- | --- | --- | --- | --- | --- | --- | --- | --- | --- | --- | --- | --- | --- | --- | --- | --- | --- | --- |
|  | |  |  |  |  | | --- | --- | --- | --- | | Job Description | AtAXR4\_\_\_ | | | | Confidence | 99.92% | Date | Tue Jan 10 14:09:39 GMT 2023 | | Rank | 70 | Aligned Residues | 267 | | % Identity | 16% | Template | c2hdwB\_ |     | PDB info | **PDB header:**hydrolase | **Chain:** B: **PDB Molecule:**hypothetical protein pa2218; | **PDBTitle:** crystal structure of hypothetical protein pa2218 from pseudomonas2 aeruginosa  **PDB Entry:** PDBe RCSB PDBj | | Resolution | 2.00 Å | | | |

Show / Hide SS confidence

Show / Hide Conservation and Alignment quality

|  |
| --- |
|  |

|  |  |  |  |  |  |  |  |  |  |
| --- | --- | --- | --- | --- | --- | --- | --- | --- | --- |
|  | Insertion relative to template ||  | Deletion relative to template |
|  | Catalytic residue from the CSA |
|  | |
| Detailed help on interpreting your alignment | |

  
  

|  |  |  |  |  |  |  |  |  |  |  |  |  |  |  |  |  |  |  |  |  |  |  |  |  |  |  |  |  |  |  |  |  |  |  |  |  |  |  |  |  |  |  |  |  |  |  |  |  |  |  |  |  |  |  |  |  |  |  |  |  |  |
| --- | --- | --- | --- | --- | --- | --- | --- | --- | --- | --- | --- | --- | --- | --- | --- | --- | --- | --- | --- | --- | --- | --- | --- | --- | --- | --- | --- | --- | --- | --- | --- | --- | --- | --- | --- | --- | --- | --- | --- | --- | --- | --- | --- | --- | --- | --- | --- | --- | --- | --- | --- | --- | --- | --- | --- | --- | --- | --- | --- | --- | --- |
|  |  | 92 | . | . | . | . | . | . | . | 100 | . | . | . | . | . | . | . | . | . | 110 |  |  |  | . | . | . | . | . | . | . | . | . | 120 | . | . | . | . | . | . | . | . | . | 130 | . | . | . |  | . | . | . | . | . | . | 140 | . | . | . | . | . | . | . |
| Predicted Secondary structure |  |  |  |  |  |  |  | --- | --- | --- | --- | --- | --- | --- |  |  |  |  |  |  | . | . | . |  | --- | --- | --- | --- | --- | --- | --- |  |  |  |  |  | --- | --- | --- | --- | --- | --- |  |  |  |  | . |  |  |  |  |  |  |  |  |  | --- | --- | --- |  |  |
| Query SS confidence |  | --- | --- | --- | --- | --- | --- | --- | --- | --- | --- | --- | --- | --- | --- | --- | --- | --- | --- | --- | . | . | . | --- | --- | --- | --- | --- | --- | --- | --- | --- | --- | --- | --- | --- | --- | --- | --- | --- | --- | --- | --- | --- | --- | --- | . | --- | --- | --- | --- | --- | --- | --- | --- | --- | --- | --- | --- | --- | --- |
| Query Sequence |  | R | T | I | K | V | Q | V | N | S | N | E | S | P | I | E | V | F | V | A | . | . | . | E | S | G | S | I | H | T | E | T | V | V | I | V | H | G | L | G | L | S | S | F | A | F | . | K | E | M | I | Q | S | L | G | S | K | G | I | H | S |
| Query Conservation |  |  |  |  |  |  |  |  |  |  |  |  |  |  | --- |  |  |  |  |  | . | . | . | --- |  | --- |  |  |  |  |  | --- | --- | --- | --- | --- | --- | --- |  |  |  | --- | --- |  |  | --- | . | --- |  | --- |  |  |  | --- | --- |  |  | --- |  | --- | --- |
| Template Conservation |  |  |  | --- |  |  | . |  | --- |  | --- | --- |  |  | --- |  |  |  |  |  |  | --- |  |  |  |  |  |  |  |  | --- |  | --- | --- |  |  | --- | --- |  |  |  |  |  |  |  |  |  |  |  |  | --- |  |  | --- | --- |  |  | --- |  |  | --- |
| Template Sequence |  | R | K | V | T | F | . | A | N | R | Y | G | I | T | L | A | A | D | L | Y | L | P | K | N | R | G | G | D | R | L | P | A | I | V | I | G | G | P | F | G | A | V | K | E | Q | S | S | G | L | Y | A | Q | T | M | A | E | R | G | F | V | T |
| Template Known Secondary structure |  |  |  |  |  |  | . |  | --- | T | T | S | --- |  |  |  |  |  |  |  |  |  | T | T | --- | --- | S | S | --- |  |  |  |  |  |  |  | --- | --- | T | T | --- | --- | T | T | T |  |  |  |  |  |  |  |  |  |  |  | T | T | --- |  |  |
| Template Predicted Secondary structure |  |  |  |  |  |  | . |  | --- | --- | --- | --- | --- |  |  |  |  |  |  |  |  | --- | --- | --- | --- | --- | --- | --- | --- | --- |  |  |  |  |  |  | --- | --- | --- | --- | --- | --- |  |  |  |  |  |  |  |  |  |  |  |  |  |  | --- | --- | --- |  |  |
| Template SS confidence |  | --- | --- | --- | --- | --- | --- | --- | --- | --- | --- | --- | --- | --- | --- | --- | --- | --- | --- | --- | --- | --- | --- | --- | --- | --- | --- | --- | --- | --- | --- | --- | --- | --- | --- | --- | --- | --- | --- | --- | --- | --- | --- | --- | --- | --- | --- | --- | --- | --- | --- | --- | --- | --- | --- | --- | --- | --- | --- | --- | --- |
|  |  | 69 | 70 | . | . | . |  | . | . | . | . | . | . | 80 | . | . | . | . | . | . | . | . | . | 90 | . | . | . | . | . | . | . | . | . | 100 | . | . | . | . | . | . | . | . | . | 110 | . | . | . | . | . | . | . | . | . | 120 | . | . | . | . | . | . | . |
|  |
|  |  | 148 | . | 150 | . | . | . | . | . | . | . | . | . | 160 | . | . | . | . | . | . | . | . | . | 170 | . | . | . | . | . | . | . | . | . | 180 | . | . | . | . | . | . | . | . | . | 190 | . | . | . | . | . | . | . | . | . | 200 | . | . | . | . | . | . | . |
| Predicted Secondary structure |  |  |  |  | --- | --- | --- | --- | --- | --- | --- | --- | --- | --- | --- | --- | --- | --- | --- | --- | --- | --- | --- | --- | --- | --- | --- | --- | --- | --- | --- | --- |  |  |  | --- | --- | --- | --- | --- | --- | --- | --- | --- | --- | --- | --- | --- | --- | --- | --- | --- | --- | --- | --- | --- | --- | --- | --- | --- | --- |
| Query SS confidence |  | --- | --- | --- | --- | --- | --- | --- | --- | --- | --- | --- | --- | --- | --- | --- | --- | --- | --- | --- | --- | --- | --- | --- | --- | --- | --- | --- | --- | --- | --- | --- | --- | --- | --- | --- | --- | --- | --- | --- | --- | --- | --- | --- | --- | --- | --- | --- | --- | --- | --- | --- | --- | --- | --- | --- | --- | --- | --- | --- | --- |
| Query Sequence |  | V | A | I | D | L | P | G | N | G | F | S | D | K | S | M | V | V | I | G | G | D | R | E | I | G | F | V | A | R | V | K | E | V | Y | G | L | I | Q | E | K | G | V | F | W | A | F | D | Q | M | I | E | T | G | D | L | P | Y | E | E | I |
| Query Conservation |  | --- | --- |  | --- | --- | --- | --- |  | --- |  | --- |  |  | --- |  |  |  |  |  |  |  |  |  |  |  |  |  |  |  |  |  |  |  |  |  |  |  |  |  |  |  |  |  |  |  |  |  |  |  |  |  |  |  |  |  |  |  |  |  |  |
| Template Conservation |  |  |  |  | --- |  | --- | --- |  | --- |  | --- |  |  |  |  |  |  |  | . | . | . | . | . | . | . | . | . | . | . | . | . | . | . | . | . | . | . | . | . | . | . | . | . | . | . | . | . | . | . | . | . | . | . | . | . | . | . | . | . | . |
| Template Sequence |  | L | A | F | D | P | S | Y | T | G | E | S | G | G | Q | P | R | N | V | . | . | . | . | . | . | . | . | . | . | . | . | . | . | . | . | . | . | . | . | . | . | . | . | . | . | . | . | . | . | . | . | . | . | . | . | . | . | . | . | . | . |
| Template Known Secondary structure |  |  |  |  | --- | --- | T | T | S | T | T | S | --- | --- | S | S | S | S | --- | . | . | . | . | . | . | . | . | . | . | . | . | . | . | . | . | . | . | . | . | . | . | . | . | . | . | . | . | . | . | . | . | . | . | . | . | . | . | . | . | . | . |
| Template Predicted Secondary structure |  |  |  |  | --- | --- | --- | --- | --- | --- | --- | --- | --- | --- | --- | --- | --- | --- | --- | . | . | . | . | . | . | . | . | . | . | . | . | . | . | . | . | . | . | . | . | . | . | . | . | . | . | . | . | . | . | . | . | . | . | . | . | . | . | . | . | . | . |
| Template SS confidence |  | --- | --- | --- | --- | --- | --- | --- | --- | --- | --- | --- | --- | --- | --- | --- | --- | --- | --- | --- | --- | --- | --- | --- | --- | --- | --- | --- | --- | --- | --- | --- | --- | --- | --- | --- | --- | --- | --- | --- | --- | --- | --- | --- | --- | --- | --- | --- | --- | --- | --- | --- | --- | --- | --- | --- | --- | --- | --- | --- | --- |
|  |  | 128 | . | 130 | . | . | . | . | . | . | . | . | . | 140 | . | . | . | . | . |  |  |  |  |  |  |  |  |  |  |  |  |  |  |  |  |  |  |  |  |  |  |  |  |  |  |  |  |  |  |  |  |  |  |  |  |  |  |  |  |  |  |
|  |
|  |  | 208 | . | 210 | . | . | . | . | . | . | . | . | . | 220 | . | . | . | . | . | . | . | . | . | 230 | . | . | . | . | . | . | . | . | . | 240 | . |  |  |  |  |  |  | . | . | . | . | . | . | . | . | 250 | . | . | . | . | . | . | . | . | . | 260 | . |
| Predicted Secondary structure |  | --- | --- | --- | --- | --- | --- | --- | --- | --- | --- | --- | --- | --- | --- | --- | --- | --- |  |  |  |  |  |  |  |  |  |  |  |  |  |  | --- | --- | --- | . | . | . | . | . | . | --- | --- |  |  |  |  |  |  | --- | --- |  |  |  |  |  |  |  |  |  |  |
| Query SS confidence |  | --- | --- | --- | --- | --- | --- | --- | --- | --- | --- | --- | --- | --- | --- | --- | --- | --- | --- | --- | --- | --- | --- | --- | --- | --- | --- | --- | --- | --- | --- | --- | --- | --- | --- | . | . | . | . | . | . | --- | --- | --- | --- | --- | --- | --- | --- | --- | --- | --- | --- | --- | --- | --- | --- | --- | --- | --- | --- |
| Query Sequence |  | I | K | L | Q | N | S | K | R | R | S | F | K | A | I | E | L | G | S | E | E | T | A | R | V | L | G | Q | V | I | D | T | L | G | L | . | . | . | . | . | . | A | P | V | H | L | V | L | H | D | S | A | L | G | L | A | S | N | W | V | S |
| Query Conservation |  |  |  |  |  |  |  |  |  |  |  |  |  |  |  |  |  |  |  |  |  |  | --- |  |  | --- |  |  |  | --- | --- |  | --- | --- | --- | . | . | . | . | . | . |  |  |  |  | --- | --- | --- | --- | --- |  | --- | --- |  | --- | --- |  |  |  | --- |  |
| Template Conservation |  | . | . | . | . | . | . | . | . | . | . | . | . | . | . | . |  |  |  |  |  |  |  |  |  |  |  |  |  |  |  |  |  |  |  |  |  |  |  |  |  |  |  |  |  |  |  | --- |  | --- |  | --- |  |  |  | --- |  |  |  | --- |  |
| Template Sequence |  | . | . | . | . | . | . | . | . | . | . | . | . | . | . | . | A | S | P | D | I | N | T | E | D | F | S | A | A | V | D | F | I | S | L | L | P | E | V | N | R | E | R | I | G | V | I | G | I | C | G | W | G | G | M | A | L | N | A | V | A |
| Template Known Secondary structure |  | . | . | . | . | . | . | . | . | . | . | . | . | . | . | . | --- | --- |  |  |  |  |  |  |  |  |  |  |  |  |  |  |  | T | T | S | T | T |  |  |  |  |  |  |  |  |  |  |  | T |  |  |  |  |  |  |  |  |  |  |  |
| Template Predicted Secondary structure |  | . | . | . | . | . | . | . | . | . | . | . | . | . | . | . | --- | --- |  |  |  |  |  |  |  |  |  |  |  |  |  |  |  | --- | --- | --- | --- | --- | --- | --- | --- | --- |  |  |  |  |  |  | --- | --- | --- |  |  |  |  |  |  |  |  |  |  |
| Template SS confidence |  | --- | --- | --- | --- | --- | --- | --- | --- | --- | --- | --- | --- | --- | --- | --- | --- | --- | --- | --- | --- | --- | --- | --- | --- | --- | --- | --- | --- | --- | --- | --- | --- | --- | --- | --- | --- | --- | --- | --- | --- | --- | --- | --- | --- | --- | --- | --- | --- | --- | --- | --- | --- | --- | --- | --- | --- | --- | --- | --- | --- |
|  |  |  |  |  |  |  |  |  |  |  |  |  |  |  |  |  | 146 | . | . | . | 150 | . | . | . | . | . | . | . | . | . | 160 | . | . | . | . | . | . | . | . | . | 170 | . | . | . | . | . | . | . | . | . | 180 | . | . | . | . | . | . | . | . | . | 190 |
|  |
|  |  | 262 | . | . | . | . | . | . | . | 270 | . | . | . | . | . | . | . | . | . | 280 | . | . | . | . | . | . | . | . | . | 290 | . | . | . | . | . | . | . | . | . | 300 | . | . | . | . | . | . | . |  |  |  |  |  |  |  |  |  |  |  |  |  | . |
| Predicted Secondary structure |  |  | --- |  |  |  |  | --- |  |  |  |  |  | --- | --- | --- | --- | --- | --- | --- | --- |  |  |  |  |  |  | --- |  |  |  |  |  |  |  |  |  | --- | --- |  |  |  |  |  |  |  |  | . | . | . | . | . | . | . | . | . | . | . | . | . |  |
| Query SS confidence |  | --- | --- | --- | --- | --- | --- | --- | --- | --- | --- | --- | --- | --- | --- | --- | --- | --- | --- | --- | --- | --- | --- | --- | --- | --- | --- | --- | --- | --- | --- | --- | --- | --- | --- | --- | --- | --- | --- | --- | --- | --- | --- | --- | --- | --- | --- | . | . | . | . | . | . | . | . | . | . | . | . | . | --- |
| Query Sequence |  | E | N | W | Q | S | V | R | S | V | T | L | I | D | S | S | I | S | P | A | L | P | L | W | V | L | N | V | P | G | I | R | E | I | L | L | A | F | S | F | G | F | E | K | L | V | S | . | . | . | . | . | . | . | . | . | . | . | . | . | F |
| Query Conservation |  |  |  | --- | --- | --- | --- |  |  | --- | --- | --- | --- | --- | --- |  |  |  |  |  |  | --- |  |  | --- |  |  |  |  |  |  |  |  |  |  |  |  |  |  |  |  |  |  |  |  | --- |  | . | . | . | . | . | . | . | . | . | . | . | . | . |  |
| Template Conservation |  |  |  |  |  |  |  |  |  |  |  |  |  |  |  |  |  |  |  |  |  |  |  |  |  |  |  |  |  |  |  |  |  |  |  |  |  |  |  |  |  |  |  |  |  |  |  |  |  |  |  |  |  |  |  |  |  |  |  |  |  |
| Template Sequence |  | V | D | K | R | V | K | A | V | V | T | S | T | M | Y | D | M | T | R | V | M | S | K | G | Y | N | D | S | V | T | L | E | Q | R | T | R | T | L | E | Q | L | G | Q | Q | R | W | K | D | A | E | S | G | T | P | A | Y | Q | P | P | Y | N |
| Template Known Secondary structure |  |  | --- | T | T |  |  |  |  |  |  |  | S | --- | --- | --- |  |  |  |  |  |  |  | T | G | G | G | --- | --- | --- |  |  |  |  |  |  |  |  |  |  |  |  |  |  |  |  |  |  |  |  |  | S | S | --- | --- | B | --- | S | --- | T | T |
| Template Predicted Secondary structure |  | --- | --- | --- | --- | --- | --- | --- | --- |  |  | --- | --- | --- | --- | --- | --- | --- | --- | --- | --- | --- | --- | --- | --- | --- | --- | --- | --- | --- | --- | --- | --- | --- |  |  |  |  |  |  | --- | --- |  |  |  |  |  |  |  | --- | --- | --- | --- | --- | --- | --- | --- | --- | --- | --- | --- |
| Template SS confidence |  | --- | --- | --- | --- | --- | --- | --- | --- | --- | --- | --- | --- | --- | --- | --- | --- | --- | --- | --- | --- | --- | --- | --- | --- | --- | --- | --- | --- | --- | --- | --- | --- | --- | --- | --- | --- | --- | --- | --- | --- | --- | --- | --- | --- | --- | --- | --- | --- | --- | --- | --- | --- | --- | --- | --- | --- | --- | --- | --- | --- |
|  |  | 191 | . | . | . | . | . | . | . | . | 200 | . | . | . | . | . | . | . | . | . | 210 | . | . | . | . | . | . | . | . | . | 220 | . | . | . | . | . | . | . | . | . | 230 | . | . | . | . | . | . | . | . | . | 240 | . | . | . | . | . | . | . | . | . | 250 |
|  |
|  |  | 309 | 310 | . | . | . | . | . | . | . | . | . | 320 | . | . | . | . | . | . | . | . | . | 330 | . | . | . | . | . | . | . | . | . | 340 | . | . | . | . | . | . | . | . | . |  |  |  |  |  | 350 | . | . | . | . | . | . | . | . | . | 360 | . | . | . |
| Predicted Secondary structure |  |  | --- | --- | --- | --- | --- | --- |  |  |  |  |  |  |  |  |  |  |  |  | --- | --- | --- | --- |  |  |  |  |  |  |  |  |  |  | --- | --- | --- | --- | --- | --- | --- |  | . | . | . | . | . |  |  |  |  |  |  | --- | --- | --- | --- | --- | --- |  |  |
| Query SS confidence |  | --- | --- | --- | --- | --- | --- | --- | --- | --- | --- | --- | --- | --- | --- | --- | --- | --- | --- | --- | --- | --- | --- | --- | --- | --- | --- | --- | --- | --- | --- | --- | --- | --- | --- | --- | --- | --- | --- | --- | --- | --- | . | . | . | . | . | --- | --- | --- | --- | --- | --- | --- | --- | --- | --- | --- | --- | --- | --- |
| Query Sequence |  | R | C | S | K | E | M | T | L | S | D | I | D | A | H | R | I | L | L | K | G | R | N | G | R | E | A | V | V | A | S | L | N | K | L | N | H | S | F | D | I | A | . | . | . | . | . | Q | W | G | N | S | D | G | I | N | G | I | P | M | Q |
| Query Conservation |  |  |  |  |  |  |  |  |  | --- |  | --- |  |  |  |  |  |  |  |  |  |  |  |  |  |  | --- |  |  |  |  |  |  |  |  |  |  |  |  |  |  |  | . | . | . | . | . |  |  |  |  |  | --- |  |  | --- |  | --- | --- | --- | --- |
| Template Conservation |  |  |  |  |  |  |  |  |  |  |  |  |  |  |  |  |  |  |  |  |  |  |  |  |  |  |  |  |  |  |  |  |  |  |  |  |  |  |  |  |  |  |  |  |  |  |  |  |  |  |  |  |  |  |  |  |  |  | --- |  | --- |
| Template Sequence |  | E | L | K | G | G | E | A | Q | F | L | V | D | Y | H | D | Y | Y | M | T | P | R | G | Y | H | P | R | A | V | N | S | G | N | A | W | T | M | T | T | P | L | S | F | M | N | M | P | I | L | T | Y | I | K | E | I | S | P | R | P | I | L |
| Template Known Secondary structure |  | --- | --- | --- | S | S | --- | --- |  |  |  |  |  |  |  |  |  |  | T | S | T | T | T | --- | --- | T | T | --- | T | T | T | T | --- | --- | --- | B | T | T | T |  |  |  |  | T | T | S | --- | S | --- | T | T | G | G | G | S | T | T | S | --- |  |  |
| Template Predicted Secondary structure |  | --- | --- | --- | --- | --- | --- | --- | --- | --- |  |  |  |  |  |  |  |  |  |  |  | --- | --- | --- | --- | --- |  |  |  | --- | --- | --- | --- | --- | --- | --- | --- | --- | --- |  |  |  |  |  |  | --- | --- | --- |  |  |  |  |  |  | --- | --- | --- | --- | --- |  |  |
| Template SS confidence |  | --- | --- | --- | --- | --- | --- | --- | --- | --- | --- | --- | --- | --- | --- | --- | --- | --- | --- | --- | --- | --- | --- | --- | --- | --- | --- | --- | --- | --- | --- | --- | --- | --- | --- | --- | --- | --- | --- | --- | --- | --- | --- | --- | --- | --- | --- | --- | --- | --- | --- | --- | --- | --- | --- | --- | --- | --- | --- | --- | --- |
|  |  | 251 | . | . | . | . | . | . | . | . | 260 | . | . | . | . | . | . | . | . | . | 270 | . | . | . | . | . | . | . | . | . | 280 | . | . | . | . | . | . | . | . | . | 290 | . | . | . | . | . | . | . | . | . | 300 | . | . | . | . | . | . | . | . | . | 310 |
|  |
|  |  | 364 | . | . | . | . | . | 370 | . | . | . | . | . | . | . | . | . | 380 | . | . | . | . | . | . | . | . | . | 390 | . | . | . | . | . |  | . | . | . | . | 400 | . | . | . | . | . | . | . |  | . | . | 410 | . | . | . | . | . | . | . |
| Predicted Secondary structure |  |  |  |  | --- | --- | --- | --- | --- | --- | --- | --- |  |  |  |  |  |  |  |  |  |  | --- | --- | --- | --- |  |  |  |  |  | --- | --- | . | --- | --- | --- | --- | --- | --- | --- | --- |  |  |  |  | . |  |  |  |  |  |  |  |  |  |  |
| Query SS confidence |  | --- | --- | --- | --- | --- | --- | --- | --- | --- | --- | --- | --- | --- | --- | --- | --- | --- | --- | --- | --- | --- | --- | --- | --- | --- | --- | --- | --- | --- | --- | --- | --- | . | --- | --- | --- | --- | --- | --- | --- | --- | --- | --- | --- | --- | . | --- | --- | --- | --- | --- | --- | --- | --- | --- | --- |
| Query Sequence |  | V | I | W | S | S | E | A | S | K | E | W | S | D | E | G | Q | R | V | A | K | A | L | P | K | A | K | F | V | T | H | S | G | . | S | R | W | P | Q | E | S | K | S | G | E | L | . | A | D | Y | I | S | E | F | V | S | L |
| Query Conservation |  | --- | --- | --- | --- |  |  | --- |  |  | --- |  |  |  |  | --- | --- |  | --- | --- |  |  | --- | --- |  | --- | --- |  | --- |  | --- |  | --- | . | --- | --- |  | --- | --- | --- | --- |  | --- |  | --- | --- | . | --- |  |  | --- |  | --- | --- | --- |  |  |
| Template Conservation |  | --- | --- |  | --- |  |  | --- |  |  |  | --- |  |  |  | . |  |  |  |  |  |  |  |  |  |  |  | --- |  |  |  |  | --- |  | --- | --- |  |  |  |  |  |  |  |  |  |  |  |  |  |  | --- |  |  | --- | --- |  |  |
| Template Sequence |  | L | I | H | G | E | R | A | H | S | R | Y | F | S | E | . | T | A | Y | A | A | A | A | E | P | K | E | L | L | I | V | P | G | A | S | H | V | D | L | Y | D | R | L | D | R | I | P | F | D | R | I | A | G | F | F | D | E |
| Template Known Secondary structure |  |  |  |  |  | T | T | --- | T | T |  |  |  |  |  | . |  |  |  |  |  | S | --- | S | S |  |  |  |  |  |  | T | T | --- | --- | S | T |  |  |  |  | --- | T | T | T | S | --- |  |  |  |  |  |  |  |  |  |  |
| Template Predicted Secondary structure |  |  |  |  |  | --- | --- | --- | --- | --- | --- |  |  |  |  | . |  |  |  |  |  | --- | --- | --- | --- | --- |  |  |  |  |  | --- | --- | --- | --- | --- |  |  |  |  | --- | --- | --- |  |  |  |  |  |  |  |  |  |  |  |  |  |  |
| Template SS confidence |  | --- | --- | --- | --- | --- | --- | --- | --- | --- | --- | --- | --- | --- | --- | --- | --- | --- | --- | --- | --- | --- | --- | --- | --- | --- | --- | --- | --- | --- | --- | --- | --- | --- | --- | --- | --- | --- | --- | --- | --- | --- | --- | --- | --- | --- | --- | --- | --- | --- | --- | --- | --- | --- | --- | --- | --- |
|  |  | 311 | . | . | . | . | . | . | . | . | 320 | . | . | . | . |  | . | . | . | . | . | 330 | . | . | . | . | . | . | . | . | . | 340 | . | . | . | . | . | . | . | . | . | 350 | . | . | . | . | . | . | . | . | . | 360 | . | . | . | . | . |
|  |

|  |  |  |
| --- | --- | --- |
| Download: | Text version | FASTA version |

No model constructed - rank, confidence too low

  

  

---

Phyre is now FREE for commercial users!

All images and data generated by Phyre2 are free to use in any
publication with acknowledgement

Accessibility Statement

|  |  |  |
| --- | --- | --- |
| **Please cite:** The Phyre2 web portal for protein modeling, prediction and analysis | | |
| Kelley LA *et al.* *Nature Protocols* 10, 845-858 (2015) [paper] [Citation link] | | |
|  | | |
| |  | | --- | | © Structural Bioinformatics Group, Imperial College, London | | Lawrence Kelley, Michael Sternberg |  | | Disclaimer | | Terms and Conditions | |  | |  | | --- | |  | | Phyre2 is part of **Genome3D** | |

  
  
