# Supplementary material for: Mechanistic insight into the role of AUXIN RESISTANCE4 in trafficking of AUXIN1 and LIKE AUX1-2
Source: Plant Physiol. 2023 Sep 30;194(1):422–33. doi: 10.1093/plphys/kiad506 (PMC10756756; doi:10.1093/plphys/kiad506)
Supplement: kiad506_Supplementary_Data [file kiad506_supplementary_data.zip › Suppemental Dataset S1/5445e4f6eaa2385e-AtAXR4/aligs/c2qtbB_.421.alig.html]

Phyre 2 alignment of AtAXR4\_\_\_ with c2qtbB\_


|  |  |
| --- | --- |
| Return to main results | Retrieve Phyre Job Id |

|  |  |  |  |  |  |  |  |  |  |  |  |  |  |  |  |  |  |  |  |  |  |  |  |  |  |
| --- | --- | --- | --- | --- | --- | --- | --- | --- | --- | --- | --- | --- | --- | --- | --- | --- | --- | --- | --- | --- | --- | --- | --- | --- | --- |
|  | |  |  |  |  | | --- | --- | --- | --- | | Job Description | AtAXR4\_\_\_ | | | | Confidence | 99.40% | Date | Tue Jan 10 14:09:39 GMT 2023 | | Rank | 421 | Aligned Residues | 206 | | % Identity | 13% | Template | c2qtbB\_ |     | PDB info | **PDB header:**hydrolase | **Chain:** B: **PDB Molecule:**dipeptidyl peptidase 4; | **PDBTitle:** human dipeptidyl peptidase iv/cd26 in complex with a 4-aryl2 cyclohexylalanine inhibitor  **PDB Entry:** PDBe RCSB PDBj | | Resolution | 2.25 Å | | | |

Show / Hide SS confidence

Show / Hide Conservation and Alignment quality

|  |
| --- |
|  |

|  |  |  |  |  |  |  |  |  |  |
| --- | --- | --- | --- | --- | --- | --- | --- | --- | --- |
|  | Insertion relative to template ||  | Deletion relative to template |
|  | Catalytic residue from the CSA |
|  | |
| Detailed help on interpreting your alignment | |

  
  

|  |  |  |  |  |  |  |  |  |  |  |  |  |  |  |  |  |  |  |  |  |  |  |  |  |  |  |  |  |  |  |  |  |  |  |  |  |  |  |  |  |  |  |  |  |  |  |  |  |  |  |  |  |  |  |  |  |  |  |  |  |  |
| --- | --- | --- | --- | --- | --- | --- | --- | --- | --- | --- | --- | --- | --- | --- | --- | --- | --- | --- | --- | --- | --- | --- | --- | --- | --- | --- | --- | --- | --- | --- | --- | --- | --- | --- | --- | --- | --- | --- | --- | --- | --- | --- | --- | --- | --- | --- | --- | --- | --- | --- | --- | --- | --- | --- | --- | --- | --- | --- | --- | --- | --- |
|  |  | 118 | . | 120 | . | . | . | . | . | . | . | . | . |  |  |  |  |  | 130 | . | . | . | . | . | . | . | . | . | 140 | . | . | . | . | . | . | . | . | . | 150 | . | . | . | . | . | . | . | . | . | 160 | . | . | . | . | . | . | . | . | . | 170 | . | . |
| Predicted Secondary structure |  | --- |  |  |  |  |  | --- | --- | --- | --- | --- | --- | . | . | . | . | . |  |  |  |  |  |  |  |  |  |  |  |  |  | --- | --- | --- |  |  |  |  |  | --- | --- | --- | --- | --- | --- | --- | --- | --- | --- | --- | --- | --- | --- | --- | --- | --- | --- | --- | --- | --- | --- |
| Query SS confidence |  | --- | --- | --- | --- | --- | --- | --- | --- | --- | --- | --- | --- | . | . | . | . | . | --- | --- | --- | --- | --- | --- | --- | --- | --- | --- | --- | --- | --- | --- | --- | --- | --- | --- | --- | --- | --- | --- | --- | --- | --- | --- | --- | --- | --- | --- | --- | --- | --- | --- | --- | --- | --- | --- | --- | --- | --- | --- | --- |
| Query Sequence |  | E | T | V | V | I | V | H | G | L | G | L | S | . | . | . | . | . | S | F | A | F | K | E | M | I | Q | S | L | G | S | K | G | I | H | S | V | A | I | D | L | P | G | N | G | F | S | D | K | S | M | V | V | I | G | G | D | R | E | I | G |
| Query Conservation |  |  | --- | --- | --- | --- | --- | --- | --- |  |  |  | --- | . | . | . | . | . | --- |  |  | --- | --- |  | --- |  |  |  | --- | --- |  |  | --- |  | --- | --- | --- | --- |  | --- | --- | --- | --- |  | --- |  | --- |  |  | --- |  |  |  |  |  |  |  |  |  |  |  |
| Template Conservation |  | --- |  | --- |  |  |  |  | --- | --- |  |  |  |  |  |  |  |  |  |  |  |  |  |  |  |  |  |  |  |  |  |  | --- |  |  | --- |  |  |  |  |  | --- | --- |  |  |  |  | --- |  |  |  |  |  |  |  |  |  |  |  |  |  |
| Template Sequence |  | P | L | L | L | D | V | Y | A | G | P | C | S | Q | K | A | D | T | V | F | R | L | N | W | A | T | Y | L | A | S | T | E | N | I | I | V | A | S | F | D | G | R | G | S | G | Y | Q | G | D | K | I | M | H | A | I | N | R | R | L | G | T |
| Template Known Secondary structure |  |  |  |  |  |  |  | --- | --- | --- | T | T | --- | --- | --- | --- | --- | --- | --- | --- | --- | --- | S |  |  |  |  |  |  |  | T | T | --- | --- |  |  |  |  |  | --- | --- | T | T | --- | S | S | S | --- |  |  |  |  | G | G | G | T | T | --- | T | T | S |
| Template Predicted Secondary structure |  |  |  |  |  |  |  | --- | --- | --- | --- | --- | --- | --- |  |  | --- | --- | --- | --- | --- | --- | --- |  |  |  |  |  |  |  | --- | --- | --- |  |  |  |  |  |  | --- | --- | --- | --- | --- | --- | --- | --- | --- |  |  |  |  |  |  |  |  | --- | --- | --- | --- |  |
| Template SS confidence |  | --- | --- | --- | --- | --- | --- | --- | --- | --- | --- | --- | --- | --- | --- | --- | --- | --- | --- | --- | --- | --- | --- | --- | --- | --- | --- | --- | --- | --- | --- | --- | --- | --- | --- | --- | --- | --- | --- | --- | --- | --- | --- | --- | --- | --- | --- | --- | --- | --- | --- | --- | --- | --- | --- | --- | --- | --- | --- | --- | --- |
|  |  | 541 | . | . | . | . | . | . | . | . | 550 | . | . | . | . | . | . | . | . | . | 560 | . | . | . | . | . | . | . | . | . | 570 | . | . | . | . | . | . | . | . | . | 580 | . | . | . | . | . | . | . | . | . | 590 | . | . | . | . | . | . | . | . | . | 600 |
|  |
|  |  | 173 | . | . | . | . | . | . | 180 | . | . | . | . | . | . | . | . | . | 190 | . | . | . | . | . | . | . | . | . | 200 | . | . | . | . | . | . | . | . | . | 210 | . | . | . | . | . | . | . | . | . | 220 | . | . | . | . | . | . | . | . | . | 230 | . | . |
| Predicted Secondary structure |  | --- | --- | --- | --- | --- | --- |  |  |  | --- | --- | --- | --- | --- | --- | --- | --- | --- | --- | --- | --- | --- | --- | --- | --- | --- | --- | --- | --- | --- | --- | --- | --- | --- | --- | --- | --- | --- | --- | --- | --- | --- | --- | --- | --- | --- | --- | --- | --- | --- | --- | --- |  |  |  |  |  |  |  |  |
| Query SS confidence |  | --- | --- | --- | --- | --- | --- | --- | --- | --- | --- | --- | --- | --- | --- | --- | --- | --- | --- | --- | --- | --- | --- | --- | --- | --- | --- | --- | --- | --- | --- | --- | --- | --- | --- | --- | --- | --- | --- | --- | --- | --- | --- | --- | --- | --- | --- | --- | --- | --- | --- | --- | --- | --- | --- | --- | --- | --- | --- | --- | --- |
| Query Sequence |  | F | V | A | R | V | K | E | V | Y | G | L | I | Q | E | K | G | V | F | W | A | F | D | Q | M | I | E | T | G | D | L | P | Y | E | E | I | I | K | L | Q | N | S | K | R | R | S | F | K | A | I | E | L | G | S | E | E | T | A | R | V | L |
| Query Conservation |  |  |  |  |  |  |  |  |  |  |  |  |  |  |  |  |  |  |  |  |  |  |  |  |  |  |  |  |  |  |  |  |  |  |  |  |  |  |  |  |  |  |  |  |  |  |  |  |  |  |  |  |  |  |  |  |  | --- |  |  | --- |
| Template Conservation |  |  | . | . | . | . | . | . | . | . | . | . | . | . | . | . | . | . | . | . | . | . | . | . | . | . | . | . | . | . | . | . | . | . | . | . | . | . | . | . | . | . | . | . | . | . | . | . | . | . | . | . |  |  |  | --- |  |  |  |  |  |
| Template Sequence |  | F | . | . | . | . | . | . | . | . | . | . | . | . | . | . | . | . | . | . | . | . | . | . | . | . | . | . | . | . | . | . | . | . | . | . | . | . | . | . | . | . | . | . | . | . | . | . | . | . | . | . | E | V | E | D | Q | I | E | A | A |
| Template Known Secondary structure |  |  | . | . | . | . | . | . | . | . | . | . | . | . | . | . | . | . | . | . | . | . | . | . | . | . | . | . | . | . | . | . | . | . | . | . | . | . | . | . | . | . | . | . | . | . | . | . | . | . | . | . |  |  |  |  |  |  |  |  |  |
| Template Predicted Secondary structure |  |  | . | . | . | . | . | . | . | . | . | . | . | . | . | . | . | . | . | . | . | . | . | . | . | . | . | . | . | . | . | . | . | . | . | . | . | . | . | . | . | . | . | . | . | . | . | . | . | . | . | . |  |  |  |  |  |  |  |  |  |
| Template SS confidence |  | --- | --- | --- | --- | --- | --- | --- | --- | --- | --- | --- | --- | --- | --- | --- | --- | --- | --- | --- | --- | --- | --- | --- | --- | --- | --- | --- | --- | --- | --- | --- | --- | --- | --- | --- | --- | --- | --- | --- | --- | --- | --- | --- | --- | --- | --- | --- | --- | --- | --- | --- | --- | --- | --- | --- | --- | --- | --- | --- | --- |
|  |  | 601 |  |  |  |  |  |  |  |  |  |  |  |  |  |  |  |  |  |  |  |  |  |  |  |  |  |  |  |  |  |  |  |  |  |  |  |  |  |  |  |  |  |  |  |  |  |  |  |  |  |  | . | . | . | . | . | . | . | . | 610 |
|  |
|  |  | 233 | . | . | . | . | . | . | 240 | . |  |  | . | . | . | . | . | . | . | . | 250 | . | . | . | . | . | . | . | . | . | 260 | . | . | . | . | . | . | . | . | . | 270 | . | . | . | . | . | . | . | . | . | 280 | . | . | . | . | . | . | . | . | . | 290 |
| Predicted Secondary structure |  |  |  |  |  |  |  | --- | --- | --- | . | . | --- | --- |  |  |  |  |  |  | --- | --- |  |  |  |  |  |  |  |  |  |  |  | --- |  |  |  |  | --- |  |  |  |  |  | --- | --- | --- | --- | --- | --- | --- | --- |  |  |  |  |  |  | --- |  |  |
| Query SS confidence |  | --- | --- | --- | --- | --- | --- | --- | --- | --- | . | . | --- | --- | --- | --- | --- | --- | --- | --- | --- | --- | --- | --- | --- | --- | --- | --- | --- | --- | --- | --- | --- | --- | --- | --- | --- | --- | --- | --- | --- | --- | --- | --- | --- | --- | --- | --- | --- | --- | --- | --- | --- | --- | --- | --- | --- | --- | --- | --- | --- |
| Query Sequence |  | G | Q | V | I | D | T | L | G | L | . | . | A | P | V | H | L | V | L | H | D | S | A | L | G | L | A | S | N | W | V | S | E | N | W | Q | S | V | R | S | V | T | L | I | D | S | S | I | S | P | A | L | P | L | W | V | L | N | V | P | G |
| Query Conservation |  |  |  |  | --- | --- |  | --- | --- | --- | . | . |  |  |  |  | --- | --- | --- | --- | --- |  | --- | --- |  | --- | --- |  |  |  | --- |  |  |  | --- | --- | --- | --- |  |  | --- | --- | --- | --- | --- | --- |  |  |  |  |  |  | --- |  |  | --- |  |  |  |  |  |
| Template Conservation |  |  |  |  |  |  |  |  |  |  | --- |  |  |  | --- |  | --- |  | --- |  | --- |  | --- | --- |  |  |  |  |  |  |  |  |  |  |  |  |  | --- |  | --- |  | --- |  |  |  |  |  |  | . | . | . | . | . | . | . | . | . | . | . | . | . |
| Template Sequence |  | R | Q | F | S | K | M | G | F | V | D | N | K | R | I | A | I | W | G | W | S | Y | G | G | Y | V | T | S | M | V | L | G | S | G | S | G | V | F | K | C | G | I | A | V | A | P | V | S | . | . | . | . | . | . | . | . | . | . | . | . | . |
| Template Known Secondary structure |  |  |  |  |  | T | T | S | S |  |  |  |  |  |  |  |  |  |  |  | T |  |  |  |  |  |  |  |  |  |  | T | T | T | --- | S | --- | --- | S |  |  |  |  |  | S | --- | --- | --- | . | . | . | . | . | . | . | . | . | . | . | . | . |
| Template Predicted Secondary structure |  |  |  |  |  |  | --- | --- | --- | --- | --- |  |  |  |  |  |  |  |  |  | --- |  |  |  |  |  |  |  |  |  |  |  |  | --- | --- | --- | --- |  |  |  |  |  |  |  | --- | --- | --- | --- | . | . | . | . | . | . | . | . | . | . | . | . | . |
| Template SS confidence |  | --- | --- | --- | --- | --- | --- | --- | --- | --- | --- | --- | --- | --- | --- | --- | --- | --- | --- | --- | --- | --- | --- | --- | --- | --- | --- | --- | --- | --- | --- | --- | --- | --- | --- | --- | --- | --- | --- | --- | --- | --- | --- | --- | --- | --- | --- | --- | --- | --- | --- | --- | --- | --- | --- | --- | --- | --- | --- | --- | --- |
|  |  | 611 | . | . | . | . | . | . | . | . | 620 | . | . | . | . | . | . | . | . | . | 630 | . | . | . | . | . | . | . | . | . | 640 | . | . | . | . | . | . | . | . | . | 650 | . | . | . | . | . | . | . |  |  |  |  |  |  |  |  |  |  |  |  |  |
|  |
|  |  | 291 | . | . | . | . | . | . | . | . | 300 | . | . | . | . | . | . | . | . | . | 310 | . | . | . | . | . | . | . | . | . | 320 | . | . | . | . | . | . | . | . | . | 330 | . | . | . | . | . | . | . | . | . | 340 | . | . | . | . | . | . | . | . | . | 350 |
| Predicted Secondary structure |  |  |  |  |  |  |  |  | --- | --- |  |  |  |  |  |  |  |  |  |  | --- | --- | --- | --- | --- | --- |  |  |  |  |  |  |  |  |  |  |  |  | --- | --- | --- | --- |  |  |  |  |  |  |  |  |  |  | --- | --- | --- | --- | --- | --- | --- |  |  |
| Query SS confidence |  | --- | --- | --- | --- | --- | --- | --- | --- | --- | --- | --- | --- | --- | --- | --- | --- | --- | --- | --- | --- | --- | --- | --- | --- | --- | --- | --- | --- | --- | --- | --- | --- | --- | --- | --- | --- | --- | --- | --- | --- | --- | --- | --- | --- | --- | --- | --- | --- | --- | --- | --- | --- | --- | --- | --- | --- | --- | --- | --- | --- |
| Query Sequence |  | I | R | E | I | L | L | A | F | S | F | G | F | E | K | L | V | S | F | R | C | S | K | E | M | T | L | S | D | I | D | A | H | R | I | L | L | K | G | R | N | G | R | E | A | V | V | A | S | L | N | K | L | N | H | S | F | D | I | A | Q |
| Query Conservation |  |  |  |  |  |  |  |  |  |  |  |  |  |  |  |  | --- |  |  |  |  |  |  |  |  |  |  | --- |  | --- |  |  |  |  |  |  |  |  |  |  |  |  |  |  | --- |  |  |  |  |  |  |  |  |  |  |  |  |  |  |  |  |
| Template Conservation |  | . | . | . | . | . | . | --- |  |  |  |  |  |  |  |  |  |  |  |  |  |  |  |  |  |  |  |  |  |  |  |  |  |  |  | . | . | . | . | . | . | . | . | . | . | . | . | . | . | . | . | . | . | . | . | . | . | . | . |  | --- |
| Template Sequence |  | . | . | . | . | . | . | R | W | E | Y | Y | D | S | V | Y | T | E | R | Y | M | G | L | P | T | P | E | D | N | L | D | H | Y | R | N | . | . | . | . | . | . | . | . | . | . | . | . | . | . | . | . | . | . | . | . | . | . | . | . | S | T |
| Template Known Secondary structure |  | . | . | . | . | . | . | --- | G | G | G | S | B |  |  |  |  |  |  |  |  | --- | --- | S | S | T | T | T | T |  |  |  |  |  |  | . | . | . | . | . | . | . | . | . | . | . | . | . | . | . | . | . | . | . | . | . | . | . | . | S | --- |
| Template Predicted Secondary structure |  | . | . | . | . | . | . | --- |  |  |  | --- | --- | --- | --- | --- | --- |  |  |  |  | --- | --- | --- | --- |  |  |  |  |  |  |  |  |  |  | . | . | . | . | . | . | . | . | . | . | . | . | . | . | . | . | . | . | . | . | . | . | . | . | --- | --- |
| Template SS confidence |  | --- | --- | --- | --- | --- | --- | --- | --- | --- | --- | --- | --- | --- | --- | --- | --- | --- | --- | --- | --- | --- | --- | --- | --- | --- | --- | --- | --- | --- | --- | --- | --- | --- | --- | --- | --- | --- | --- | --- | --- | --- | --- | --- | --- | --- | --- | --- | --- | --- | --- | --- | --- | --- | --- | --- | --- | --- | --- | --- | --- |
|  |  |  |  |  |  |  |  | 658 | . | 660 | . | . | . | . | . | . | . | . | . | 670 | . | . | . | . | . | . | . | . | . | 680 | . | . | . | . | . |  |  |  |  |  |  |  |  |  |  |  |  |  |  |  |  |  |  |  |  |  |  |  |  | . | . |
|  |
|  |  | 351 | . | . | . | . | . | . | . |  | . | 360 | . | . | . | . | . | . | . | . | . | 370 | . | . | . | . | . | . | . | . | . | 380 | . | . | . | . | . | . | . |  |  |  |  | . | . | 390 | . | . | . | . | . |  | . | . | . | . | 400 | . |  | . | . |
| Predicted Secondary structure |  |  |  |  |  |  | --- | --- | --- | . | --- | --- | --- |  |  |  |  |  | --- | --- | --- | --- | --- | --- | --- | --- |  |  |  |  |  |  |  |  |  |  | --- | --- | --- | . | . | . | . | --- |  |  |  |  |  | --- | --- | . | --- | --- | --- | --- | --- | --- | . | --- | --- |
| Query SS confidence |  | --- | --- | --- | --- | --- | --- | --- | --- | . | --- | --- | --- | --- | --- | --- | --- | --- | --- | --- | --- | --- | --- | --- | --- | --- | --- | --- | --- | --- | --- | --- | --- | --- | --- | --- | --- | --- | --- | . | . | . | . | --- | --- | --- | --- | --- | --- | --- | --- | . | --- | --- | --- | --- | --- | --- | . | --- | --- |
| Query Sequence |  | W | G | N | S | D | G | I | N | . | G | I | P | M | Q | V | I | W | S | S | E | A | S | K | E | W | S | D | E | G | Q | R | V | A | K | A | L | P | K | . | . | . | . | A | K | F | V | T | H | S | G | . | S | R | W | P | Q | E | . | S | K |
| Query Conservation |  |  |  |  |  | --- |  |  | --- | . |  | --- | --- | --- | --- | --- | --- | --- | --- |  |  | --- |  |  | --- |  |  |  |  | --- | --- |  | --- | --- |  |  | --- | --- |  | . | . | . | . | --- | --- |  | --- |  | --- |  | --- | . | --- | --- |  | --- | --- | --- | . | --- |  |
| Template Conservation |  | --- |  |  |  |  |  |  |  |  |  |  | --- |  | --- |  |  |  | --- |  |  | --- |  |  | --- |  |  |  |  |  |  |  |  |  |  |  | --- |  |  |  | --- |  |  |  |  |  |  |  |  |  |  |  |  | --- |  |  |  |  |  |  |  |
| Template Sequence |  | V | M | S | R | A | E | N | F | K | Q | V | E | Y | L | L | I | H | G | T | A | D | D | N | V | H | F | Q | Q | S | A | Q | I | S | K | A | L | V | D | V | G | V | D | F | Q | A | M | W | Y | T | D | E | D | H | G | I | A | S | S | T | A |
| Template Known Secondary structure |  | S | G | G | G | G | G | G | G | G | G | S |  |  |  |  |  |  |  | T | T | --- | S | S | S | --- | T |  |  |  |  |  |  |  |  |  |  |  |  | T | T | --- | --- | --- |  |  |  |  |  | T | T | --- | --- | T | T | --- | --- | S |  |  |  |
| Template Predicted Secondary structure |  |  |  |  |  |  |  |  | --- | --- | --- | --- | --- |  |  |  |  |  | --- | --- | --- | --- | --- | --- | --- | --- |  |  |  |  |  |  |  |  |  |  |  |  |  | --- | --- | --- | --- |  |  |  |  |  |  | --- | --- | --- | --- | --- | --- | --- | --- | --- | --- | --- |  |
| Template SS confidence |  | --- | --- | --- | --- | --- | --- | --- | --- | --- | --- | --- | --- | --- | --- | --- | --- | --- | --- | --- | --- | --- | --- | --- | --- | --- | --- | --- | --- | --- | --- | --- | --- | --- | --- | --- | --- | --- | --- | --- | --- | --- | --- | --- | --- | --- | --- | --- | --- | --- | --- | --- | --- | --- | --- | --- | --- | --- | --- | --- | --- |
|  |  | 688 | . | 690 | . | . | . | . | . | . | . | . | . | 700 | . | . | . | . | . | . | . | . | . | 710 | . | . | . | . | . | . | . | . | . | 720 | . | . | . | . | . | . | . | . | . | 730 | . | . | . | . | . | . | . | . | . | 740 | . | . | . | . | . | . | . |
|  |
|  |  | 404 | . | . | . | . | . | 410 | . | . | . | . | . | . |
| Predicted Secondary structure |  |  |  |  |  |  |  |  |  |  |  |  |  |  |
| Query SS confidence |  | --- | --- | --- | --- | --- | --- | --- | --- | --- | --- | --- | --- | --- |
| Query Sequence |  | S | G | E | L | A | D | Y | I | S | E | F | V | S |
| Query Conservation |  | --- |  | --- | --- | --- |  |  | --- |  | --- | --- | --- |  |
| Template Conservation |  |  |  |  |  |  |  |  |  |  |  | --- |  |  |
| Template Sequence |  | H | Q | H | I | Y | T | H | M | S | H | F | I | K |
| Template Known Secondary structure |  |  |  |  |  |  |  |  |  |  |  |  |  |  |
| Template Predicted Secondary structure |  |  |  |  |  |  |  |  |  |  |  |  |  |  |
| Template SS confidence |  | --- | --- | --- | --- | --- | --- | --- | --- | --- | --- | --- | --- | --- |
|  |  | 748 | . | 750 | . | . | . | . | . | . | . | . | . | 760 |
|  |

|  |  |  |
| --- | --- | --- |
| Download: | Text version | FASTA version |

No model constructed - rank, confidence too low

  

  

---

Phyre is now FREE for commercial users!

All images and data generated by Phyre2 are free to use in any
publication with acknowledgement

Accessibility Statement

|  |  |  |
| --- | --- | --- |
| **Please cite:** The Phyre2 web portal for protein modeling, prediction and analysis | | |
| Kelley LA *et al.* *Nature Protocols* 10, 845-858 (2015) [paper] [Citation link] | | |
|  | | |
| |  | | --- | | © Structural Bioinformatics Group, Imperial College, London | | Lawrence Kelley, Michael Sternberg |  | | Disclaimer | | Terms and Conditions | |  | |  | | --- | |  | | Phyre2 is part of **Genome3D** | |

  
  
